# Exploring the common mechanisms and biomarker ST8SIA4 of atherosclerosis and ankylosing spondylitis through bioinformatics analysis and machine learning

**DOI:** 10.3389/fcvm.2024.1421071

**Published:** 2024-07-18

**Authors:** Yirong Ma, Junyu Lai, Qiang Wan, Liqiang Sun, Yang Wang, Xingliang Li, Qinhe Zhang, Jianguang Wu

**Affiliations:** ^1^ Department of Postgraduate, Jiangxi University of Traditional Chinese Medicine, Nanchang, China; ^2^ Cardiology Department, Affiliated Hospital of Jiangxi University of Traditional Chinese Medicine, Nanchang, China; ^3^ Department of Acupuncture and Tuina, Jiangxi University of Traditional Chinese Medicine, Nanchang, China

**Keywords:** atherosclerosis, ankylosing spondylitis, WGCNA, machine learning algorithm, ST8SIA4

## Abstract

**Background:**

Atherosclerosis (AS) is a major contributor to cerebrovascular and cardiovascular events. There is growing evidence that ankylosing spondylitis is closely linked to AS, often co-occurring with it; however, the shared pathogenic mechanisms between the two conditions are not well understood. This study employs bioinformatics approaches to identify common biomarkers and pathways between AS and ankylosing spondylitis.

**Methods:**

Gene expression datasets for AS (GSE100927, GSE28829, GSE155512) and ankylosing spondylitis (GSE73754, GSE25101) were obtained from the Gene Expression Omnibus (GEO). Differential expression genes (DEGs) and module genes for AS and ankylosing spondylitis were identified using the Limma R package and weighted gene co-expression network analysis (WGCNA) techniques, respectively. The machine learning algorithm SVM-RFE was applied to pinpoint promising biomarkers, which were then validated in terms of their expression levels and diagnostic efficacy in AS and ankylosing spondylitis, using two separate GEO datasets. Furthermore, the interaction of the key biomarker with the immune microenvironment was investigated via the CIBERSORT algorithm, single-cell analysis was used to identify the locations of common diagnostic markers.

**Results:**

The dataset GSE100927 contains 524 DEGs associated with AS, whereas dataset GSE73754 includes 1,384 genes categorized into modules specific to ankylosing spondylitis. Analysis of these datasets revealed an overlap of 71 genes between the DEGs of AS and the modular genes of ankylosing spondylitis. Utilizing the SVM-RFE algorithm, 15 and 24 central diagnostic genes were identified in datasets GSE100927 and GSE73754, respectively. Further validation of six key genes using external datasets confirmed ST8SIA4 as a common diagnostic marker for both conditions. Notably, ST8SIA4 is upregulated in samples from both diseases. Additionally, ROC analysis confirmed the robust diagnostic utility of ST8SIA4. Moreover, analysis through CIBERSORT suggested an association of the ST8SIA4 gene with the immune microenvironment in both disease contexts. Single-cell analysis revealed that ST8SIA4 is primarily expressed in Macrophages, Monocytes, T cells, and CMPs.

**Conclusion:**

This study investigates the role of ST8SIA4 as a common diagnostic gene and the involvement of the lysosomal pathway in both AS and ankylosing spondylitis. The findings may yield potential diagnostic biomarkers and offer new insights into the shared pathogenic mechanisms underlying these conditions.

## 1 Introduction

Atherosclerosis (AS) serves as the foundational pathological mechanism for cardiovascular diseases, which rank prominently among the leading causes of disability and mortality globally (1). According to the Global Burden of Disease Study, the incidence of cardiovascular diseases saw a significant escalation rising from 271 million instances in 1990 to 523 million in 2019. Concurrently, fatalities linked to these conditions climbed from 12.1 million to 18.6 million within the same timeframe (2). AS develops from the accumulation of fibro-fatty lesions in arterial walls, accompanied by the infiltration of immune cells such as macrophages, T cells, and mast cells and is characterized as a chronic inflammatory condition with autoimmune features, influenced by complex interactions among immune metabolic alterations and oxidative stress (3). Spondyloarthritis (SPA), encompassing a range of inflammatory diseases affecting the spine, peripheral joints, and synovium, is categorized into peripheral spondyloarthritis and ankylosing spondylitis, with the latter further divided into radiographic axial spondyloarthritis (also known as ankylosing spondylitis) and non-radiographic axial spondyloarthritis (4). Ankylosing spondylitis, the second most prevalent form of spondylitis, predominantly impacts the spine, peripheral joints, ligaments, and tendons, and is associated with a heightened cardiovascular risk (4, 5). Research indicates that ankylosing spondylitis may increase the risk of AS leading to a 50%–100% higher incidence of cardiovascular diseases in these patients (6–9). Inflammation in ankylosing spondylitis directly compromises vascular structures and exacerbates AS by affecting cardiovascular risk factors such as lipid levels, blood pressure, and insulin resistance (6, 10). In one study, 39% of Psoriatic arthritis (PsA) patients exhibited carotid plaques during ultrasound examinations (11). A smaller study using coronary computed tomography angiography revealed a 76% prevalence of coronary artery plaques in PsA patients, compared to 44% in the control group (12). PsA and ankylosing spondylitis, as distinct forms of inflammatory arthritis, share genetic backgrounds, immunological responses, and clinical treatment approaches, thereby demonstrating a significant interconnection (13). Further, Ozdowska et al. reported a higher occurrence of subclinical coronary AS in patients with ankylosing spondylitis (14).

Although the intrinsic connection mechanisms between AS and ankylosing spondylitis are not fully understood, it is evident that inflammatory responses and abnormal activation of the immune system play significant roles in both diseases.The pathogenesis of AS primarily involves the subendothelial deposition of low-density lipoprotein (LDL), which triggers a local inflammatory response. Subsequently, macrophages that ingest LDL transform into foam cells, further intensifying inflammation, leading to endothelial damage and hardening of the arterial walls (15). Ankylosing spondylitis is typically understood as an autoimmune disorder where the immune system erroneously targets the body's own tissues, especially affecting the spine and pelvic joints. Research shows that immune cells, including T cells and macrophages, play a role in this process by targeting these areas and discharging inflammatory mediators, which in turn leads to symptoms such as pain, swelling, and stiffness of the joints (16). Furthermore, the advancement of both AS and ankylosing spondylitis is linked to the synthesis of various cytokines and inflammatory mediators. Notably, increased concentrations of CRP (C-reactive protein), TNF-α (tumor necrosis factor-alpha), and IL-6 (interleukin-6) have been observed in these diseases, playing pivotal roles in the enhancement of inflammatory responses and the progression of disease-related pathologies (17–19). The similar expression patterns of these cytokines reflect the similarity in the immune-inflammatory responses of the two diseases. Thus, the detection of immune infiltration and associated inflammatory molecules could offer diagnostic advantages for patients with AS combine with ankylosing spondylitis, which is essential for preventing severe cardiovascular outcomes.

**Figure 1:**
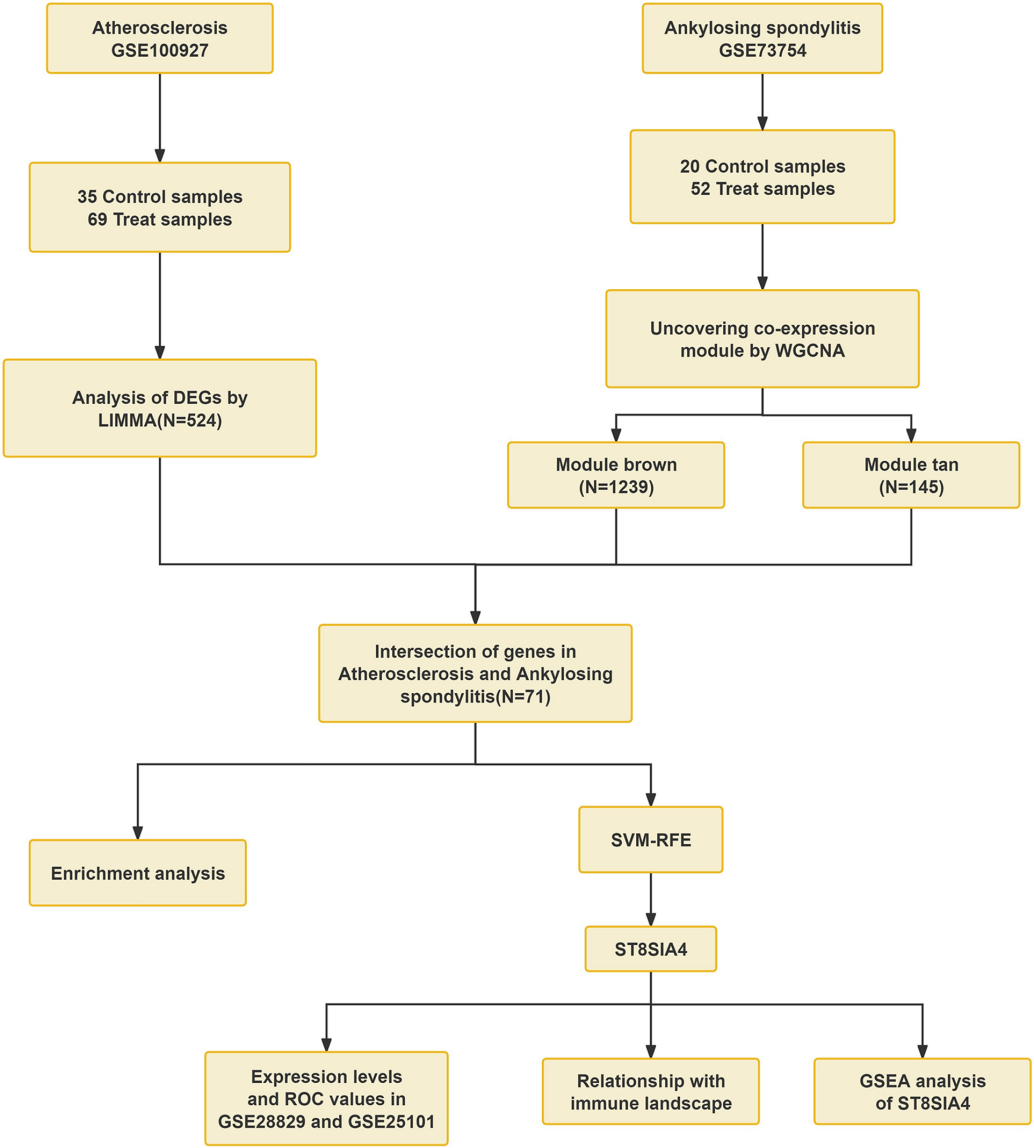
Workflow of the analysis.

Currently, genetic studies investigating the association between AS and ankylosing spondylitis are limited. This study aims to identify common biomarkers and elucidate the molecular mechanisms underlying AS and ankylosing spondylitis. We evaluated mRNA expression data from the GEO database. Through employing weighted gene co-expression network analysis (WGCNA) and machine learning technique, ST8SIA4 was pinpointed as a key diagnostic biomarker for both conditions. Furthermore, Gene set enrichment analysis (GSEA) revealed potential involvement of the lysosomal pathway in their pathology. Analysis using CIBERSORT also indicated that ST8SIA4 is implicated in the alterations of the immune microenvironment linked to these disorders (Figure 1). Overall, our findings spotlight ST8SIA4 as a critical diagnostic gene and the lysosomal pathway as a common pathway in AS and ankylosing spondylitis, aiming to foster novel diagnostic and therapeutic approaches for these conditions.

## 2 Materials and methods

### 2.1 Data collection and data processing

We obtained gene expression profile datasets along with clinical data from the GEO database by conducting searches with the terms “atherosclerosis” and “ankylosing spondylitis” for microarray datasets. To ensure the accuracy of WGCNA, we excluded non-human specimens and selected sample groups containing a minimum of 15 samples. The final datasets obtained included GSE100927, GSE73754, GSE28829, GSE25101 and GSE155512. The GSE100927 dataset, utilized on the GPL17077-17467 platform, included tissue samples from 35 healthy controls and 69 patients with AS. The GSE73754 dataset, employed on the GPL10558 platform, consisted of peripheral blood mononuclear cells (PBMCs) from 52 patients with ankylosing spondylitis and 20 healthy controls. Furthermore, the dataset GSE28829, which relies on the GPL570 platform, including carotid artery plaque samples from 16 late-stage and 13 early-stage atherosclerosis patients, served as an external validation set. In a similar manner, the GSE25101 dataset, based on the GPL6947 platform, including gene expression data from whole blood of 16 patients with ankylosing spondylitis and 16 gender and age-matched healthy controls, used as an external validation set. The GSE155512 dataset includes tissue samples from three individuals who underwent carotid endarterectomy. These samples were procured from atherosclerotic carotid arteries, from which single cells were isolated using the 10× Genomics Chromium Single Cell Gene Expression system. Subsequently, these cells were analyzed through RNA sequencing.

### 2.2 Differential gene expression screening

Differentially expressed genes (DEGs) in the GSE100927 dataset were identified employing the “Limma” R package. Criteria for screening included |logFC|>1 and adjusted *P*-value <0.05. For visualization, the “ggplot2” package was used to create volcano plots and heatmaps for the top 50 genes with differential expression rankings.

### 2.3 Weighted gene coexpression network analysis

The analytical framework known as weighted gene co-expression network analysis (WGCNA) was applied to identify significant clusters of co-expressed genes and to elucidate the connections between gene networks and pathological conditions. In our research, the WGCNA R package was harnessed to discern correlations between gene expressions and phenotypic traits by establishing a gene co-expression network (20). Initially, genes displaying the bottom 50% in terms of median absolute deviation (MAD) were omitted. Following this, the Pearson correlation coefficients were computed for all gene pairs, and a weighted adjacency matrix was formulated using average linkage along with weighted correlation coefficients. A soft thresholding power (b) was then applied to compute adjacency, which was further transformed into a Topological Overlap Matrix (TOM). For the purpose of clustering genes that exhibit similar expression patterns, average linkage hierarchical clustering was executed using TOM-based dissimilarity, setting the smallest module size at 60 genes. The process culminated in evaluating the congruence of hub genes across modules, establishing a cutoff for the module dendrogram, and amalgamating multiple modules. This approach was specifically employed to pinpoint crucial modules in the context of ankylosing spondylitis and to produce visual representations of the pertinent gene networks.

### 2.4 Functional enrichment analysis

To investigate the biological roles of genes, we employed the “clusterProfiler” package in R. Our initial step involved performing Gene Ontology (GO) and Kyoto Encyclopedia of Genes and Genomes (KEGG) analyses, recognizing pathways as significant if they presented with a *P*-value <0.05 (21, 22). We then proceeded to ascertain the genes common between the DEGs in AS and the principal module genes in ankylosing spondylitis, conducting additional GO and KEGG analyses on these shared genes. Visualization of the results was facilitated through the use of the “ggplot2” package.

### 2.5 Identification and verification of diagnostic biomarkers

To pinpoint potential diagnostic markers for AS with ankylosing spondylitis, we utilized the Support Vector Machine Recursive Feature Elimination (SVM-RFE) technique. This method, facilitated by the “e1071” package in R (23), strategically removes feature vectors to isolate key genes that may serve diagnostic purposes. We corroborated the relevance of these genes using datasets GSE28829 and GSE25101. Further, we explored the associations between these identified genes and specific immune cells within the datasets to establish their significant linkages to immune cell activities. For evaluating the diagnostic accuracy of our model, we calculated the area under the receiver operating characteristic (ROC) curve (AUC) using the “pROC” package in R, which measures the effectiveness of the identified core genes.

### 2.6 Gene set enrichment analysis

We conducted single-gene Gene Set Enrichment Analysis (GSEA) employing the “ClusterProfiler” package in R to discern pathway-level distinctions between disease and control cohorts. The gene set utilized for GSEA, designated as c2.cp.kegg.v7.5.1.symbols.gmt, was sourced from the MSigDB database (24). Only KEGG pathways demonstrating a *P*-value <0.05 were deemed statistically significant. We displayed the outcomes using the “enrichplot” package in R.

### 2.7 Immune analysis algorithm

To investigate immune cell infiltration in AS and ankylosing spondylitis samples, we applied the CIBERSORT deconvolution algorithm, designed to estimate the proportions of 22 distinct immune cell types using gene expression data. The analysis was conducted on the datasets GSE73754 and GSE100927 (25). We visualized the outcomes employing the R packages “corrplot”, “ggplot2”, and “ggpubr”. Furthermore, we utilized the non-parametric Spearman correlation method to assess the associations between core biomarkers and the expression of immune-infiltrating cells.

### 2.8 Single-cell sequencing analysis

We downloaded the AS scRNA-seq dataset GSE155512 from GEO. Downstream analysis was performed using the Seurat R package (26). Initially, a Seurat object was created from the single-cell expression data, and quality control was applied, excluding cells with fewer than 50 expressed genes or mitochondrial gene expression exceeding 5%. The data was normalized using the “LogNormalize” method, and 1,500 highly variable genes were identified with the “FindVariableFeatures” function. Subsequently, principal component analysis (PCA), cluster analysis using Seurat's “FindClusters” function, and t-SNE (t-distributed stochastic neighbor embedding) were conducted for non-linear dimensionality reduction, illustrating the results in t-SNE.

## 3 Results

### 3.1 Identification of differentially expressed genes

**Figure 2:**
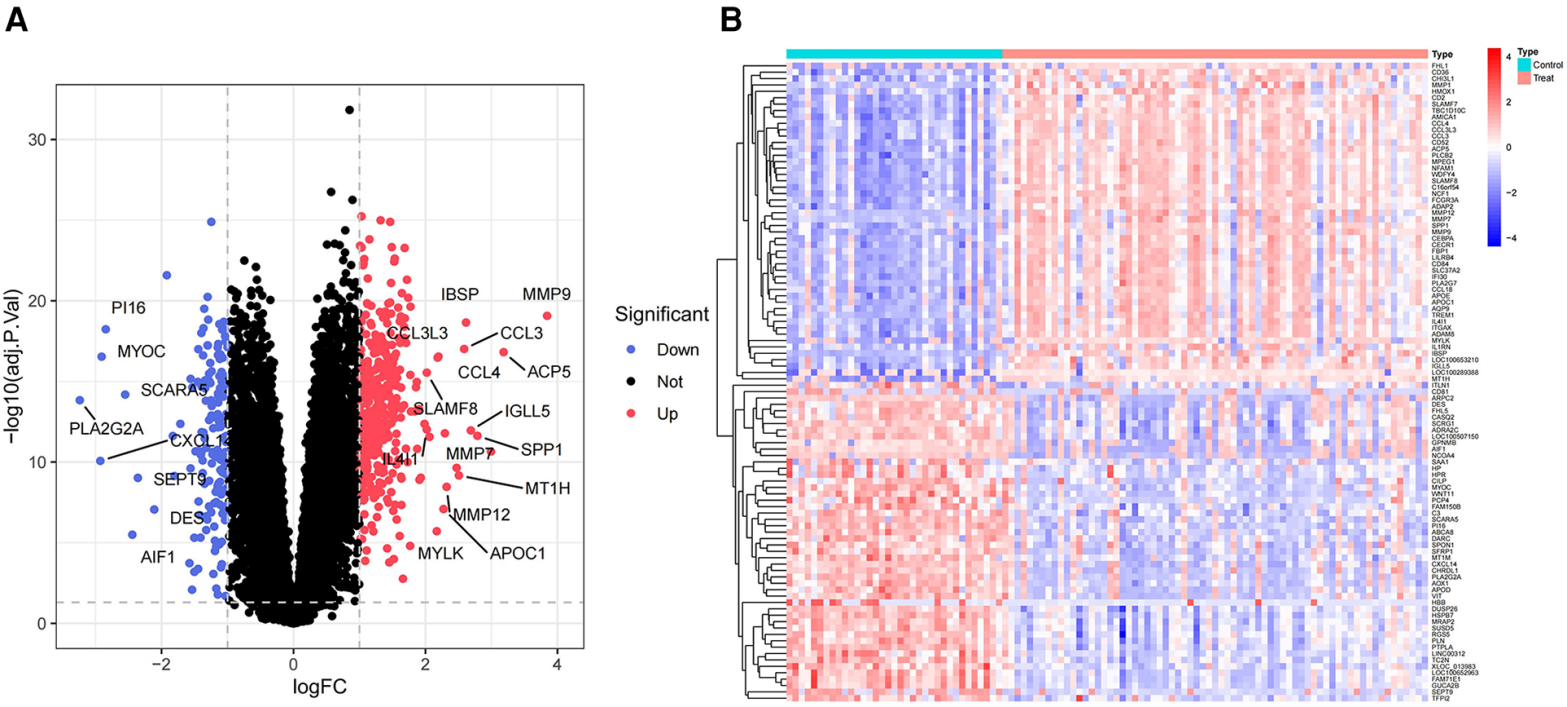
Volcano plot and heatmap of the DEGs identified from GSE100927. (**A**) Volcano map of DEGs from GSE100927. (**B**) Heatmap of DEGs from GSE100927.

This analysis compared the gene expression profiles of patients with atherosclerosis and healthy controls in the GSE100927 dataset. DEG analysis revealed 524 genes exhibiting differential expression within the dataset pertaining to AS. Among these, 363 genes were found to be upregulated, while 161 genes were downregulated. Volcano plots for all differentially expressed genes and heatmaps for the top 50 most significantly expressed genes were generated using R (Figures 2A,B).

### 3.2 Weighted gene co-expression network analysis and identification of key modules

**Figure 3:**
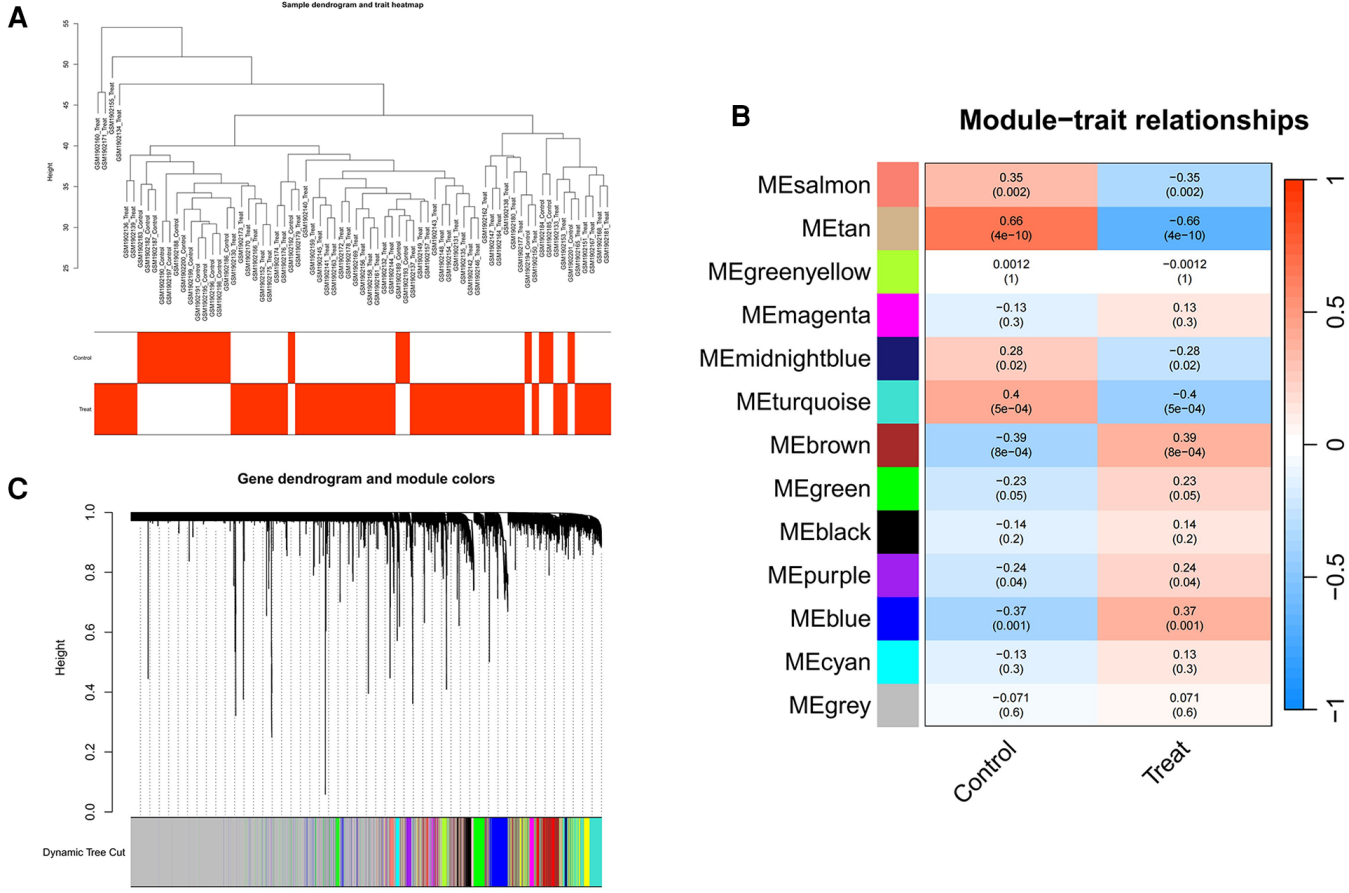
Screening of genes in the GSE73754 datasets using the WGCNA algorithm. (**A**) Clustering dendrogram of samples. (**B**) The correlation coefficients between gene modules and ankylosing spondylitis. (**C**) Dendrogram of the gene modules. WGCNA, weighted gene coexpression network analysis.

We employed WGCNA to develop a scale-free co-expression network and pinpoint modules strongly associated with ankylosing spondylitis. For the GSE73754 dataset, a “soft” threshold of *b* = 13 was adopted, predicated on achieving scale independence and optimal average connectivity (Figure 3A; Supplementary Data Sheet 1). WGCNA revealed 13 modules. Clinical correlation analysis demonstrated that the “MEbrown” and “MEtan” modules exhibited the highest positive and negative correlations with ankylosing spondylitis, respectively (MEbrown: *r* = 0.39, *p* = 8e-04; MEtan: *r* = −0.66, *p* = 4e-10) (Figures 3B,C). Consequently, we selected the brown module, which comprises 1,239 genes, and the tan module, which comprises 145 genes, for further analysis.

### 3.3 Identification of shared genes and pathways

**Figure 4:**
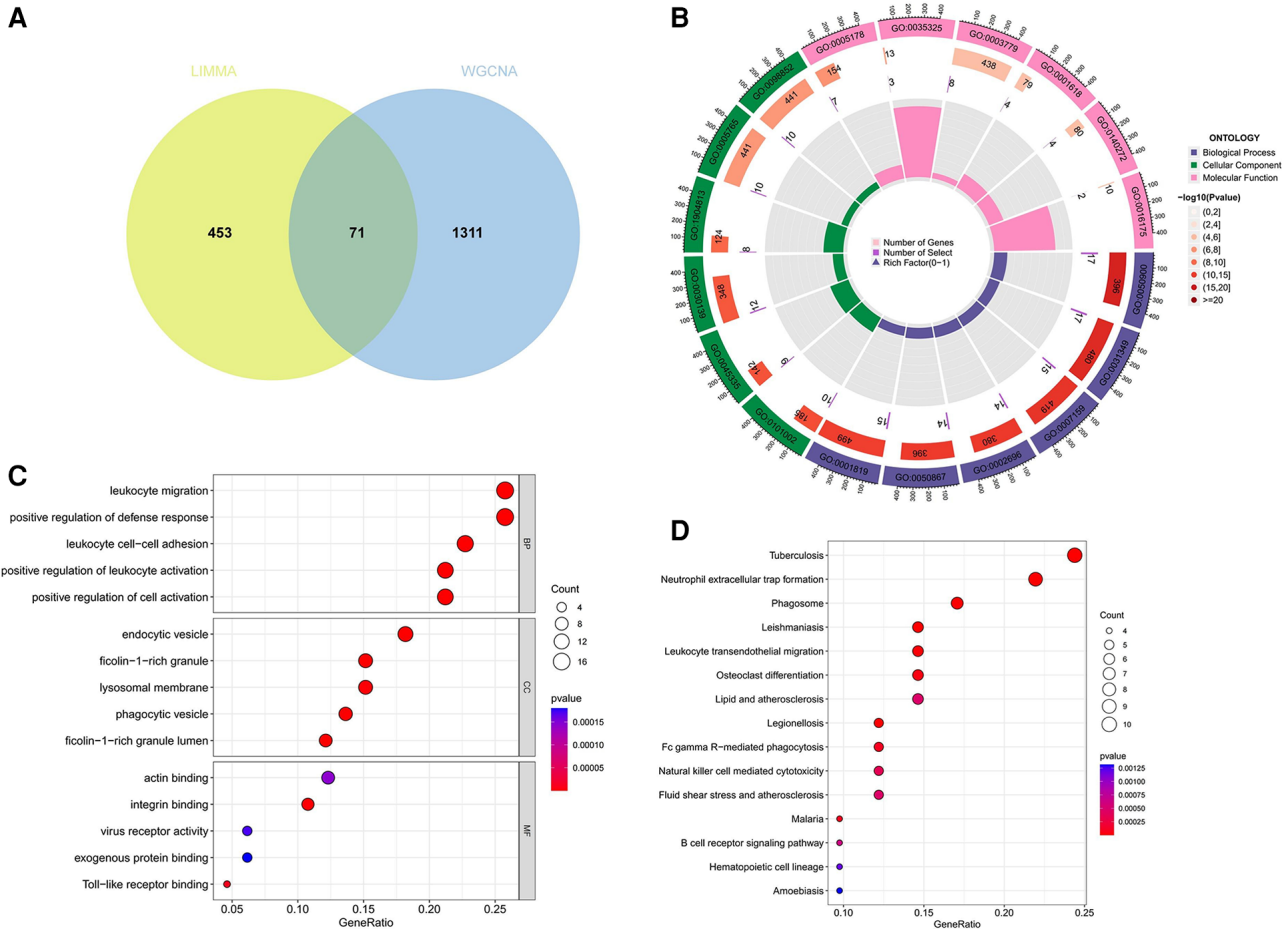
Function enrichment analysis of the intersecting genes. (**A**) The intersecting of DEGs via Limma and WGCNA module genes includes 71 genes, which were shown in the Veen diagram. (**B,C**) Circle plot and bubble plot of GO enrichment analysis includes biological process, cellular component and molecular function. (**D**) Bubble plot of KEGG enrichment. GO, gene ontology; KEGG, Kyoto encyclopedia of genes and genomes.

A total of 71 genes have been discovered that coincide between the DEGs linked to AS and the key modules relevant to ankylosing spondylitis (Figure 4A; Supplementary Data Sheet 2). It is suggested that these genes might play a role in the development of both AS and ankylosing spondylitis. Enrichment analysis conducted on these genes indicated significant associations. GO analysis revealed that the biological process (BP) genes are predominantly involved in leukocyte migration, positive regulation of the defense response, leukocyte cell-cell adhesion, and positive regulation of leukocyte activation (Figures 4B,C). Cellular component (CC) genes are enriched in ficolin-1-rich granules, endocytic vesicles, phagocytic vesicles, and lysosomal membranes. Molecular function (MF) genes are enriched in functions such as integrin binding, Toll-like receptor binding, and actin binding. Furthermore, KEGG pathway analysis shows significant enrichment in pathways related to tuberculosis, neutrophil extracellular trap formation, phagosomes, leishmaniasis, and leukocyte transendothelial migration (Figure 4D).

### 3.4 Identification and validation of potential shared diagnostic biomarkers

**Figure 5:**
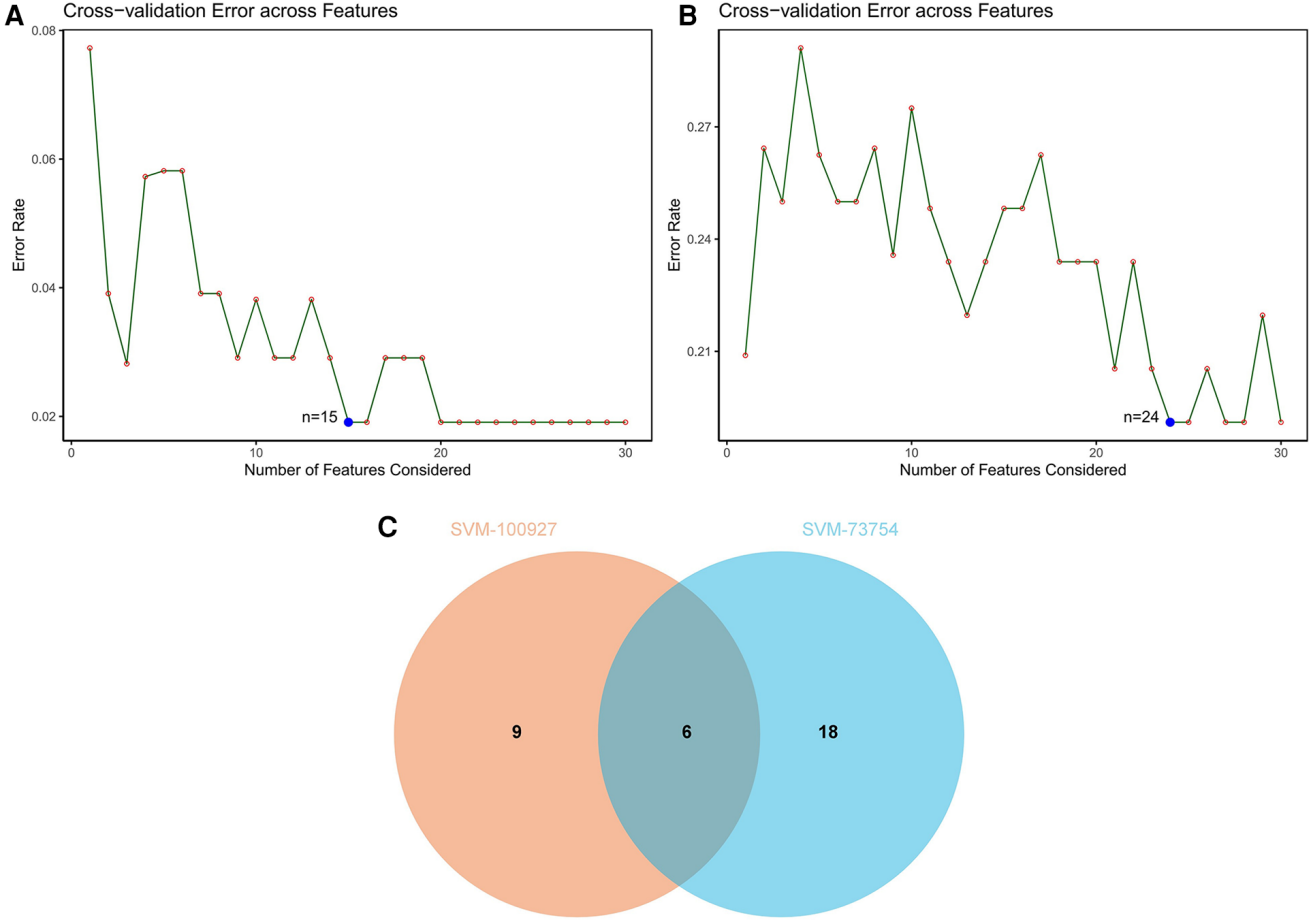
Identification of diagnostic genes using SVM-RFE algorithm. (**A**) Feature genes selection in GSE100927. (**B**) Feature genes selection in GSE73754. (**C**) Venn diagram of overlapping feature genes.

**Figure 6:**
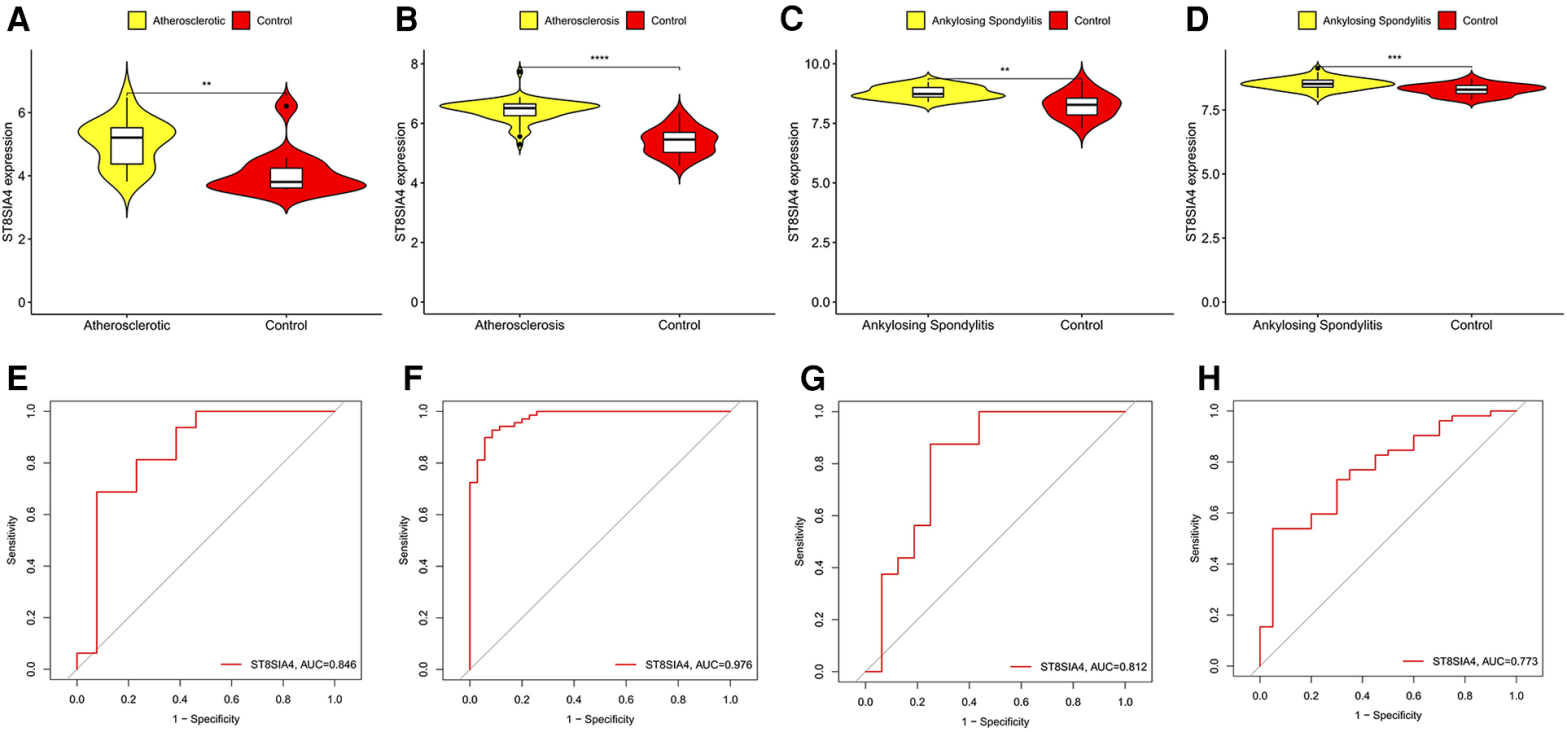
Validation of the expression level and diagnostic efficacy of ST8SIA4 gene. The violin plots of ST8SIA4 gene in GSE28829 (**A**), GSE100927 (**B**), GSE25101 (**C**) and GSE73754 (**D**) The ROC curves of ST8SIA4 gene in GSE28829 (**E**), GSE100927 (**F**), GSE25101 (**G**) and GSE73754 (**H**) *p < 0.05; **p < 0.01;***p < 0.001; ****p < 0.0001.

SVM-RFE is a machine learning approach derived from Support Vector Machines, designed to identify optimal core genes by filtering out feature vectors produced by the SVM. Analysis of 71 common genes revealed 15 potential central diagnostic genes in GSE100927 (Figure 5A), and 24 in GSE73754 (Figure 5B). After intersecting the datasets, six core genes were identified (Figure 5C). Validation using external datasets revealed no significant differences for CSF3R, CTSS, MNDA, and TLR2. Furthermore, it was noted that the CYTH4 gene was not present in the GSE25101 dataset. Ultimately, ST8SIA4 was confirmed as the most likely optimal diagnostic biomarker for AS combine with ankylosing spondylitis (Supplementary Data Sheet 3). Subsequent verification across four datasets showed that ST8SIA4 expression levels were consistently higher in the AS or ankylosing spondylitis groups compared to healthy controls (Figures 6A–D). Furthermore, ROC analysis demonstrated significant diagnostic efficacy of ST8SIA4, with AUC values of 0.976 in GSE100927 and 0.773 in GSE73754 (Figures 6F,H). In further independent analyses, ST8SIA4 demonstrated significant diagnostic effectiveness in datasets GSE28829 and GSE25101, achieving AUC values of 0.846 and 0.812, respectively (Figures 6E,G).

### 3.5 GSEA analysis

**Figure 7:**
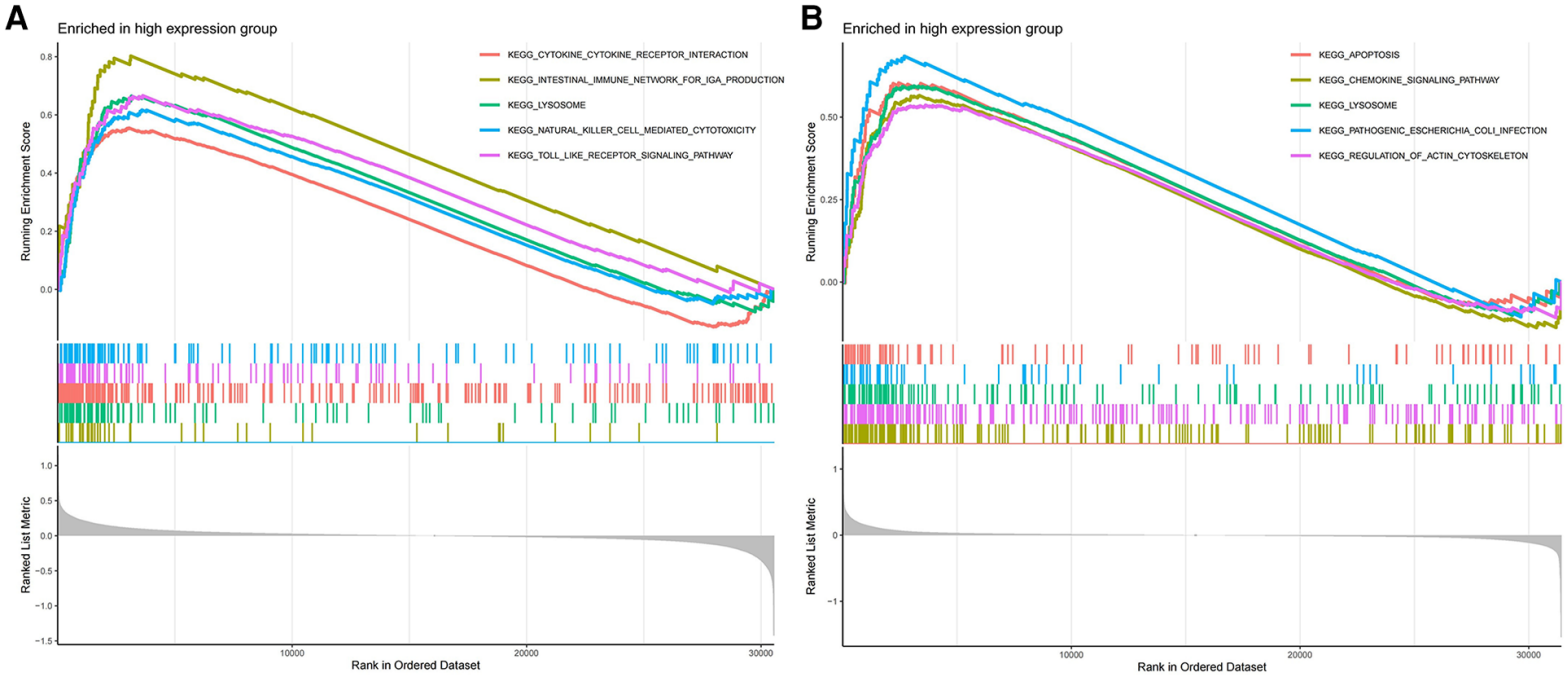
(**A**) GSEA analyses results of aS samples. (**B**) GSEA analyses results of ankylosing spondylitis samples.

The GSEA results demonstrate that in AS samples, the lysosomal, cytokine receptor interaction, and toll-like receptor signaling pathways are positively enriched (Figure 7A). Furthermore, in samples from ankylosing spondylitis, positive enrichment is also evident in the lysosomal and chemokine signaling pathways, as well as in response to pathogenic Escherichia coli infection (Figure 7B). Notably, the lysosomal pathway is consistently enriched across both conditions.

### 3.6 Immunocellular infiltration analysis

**Figure 8:**
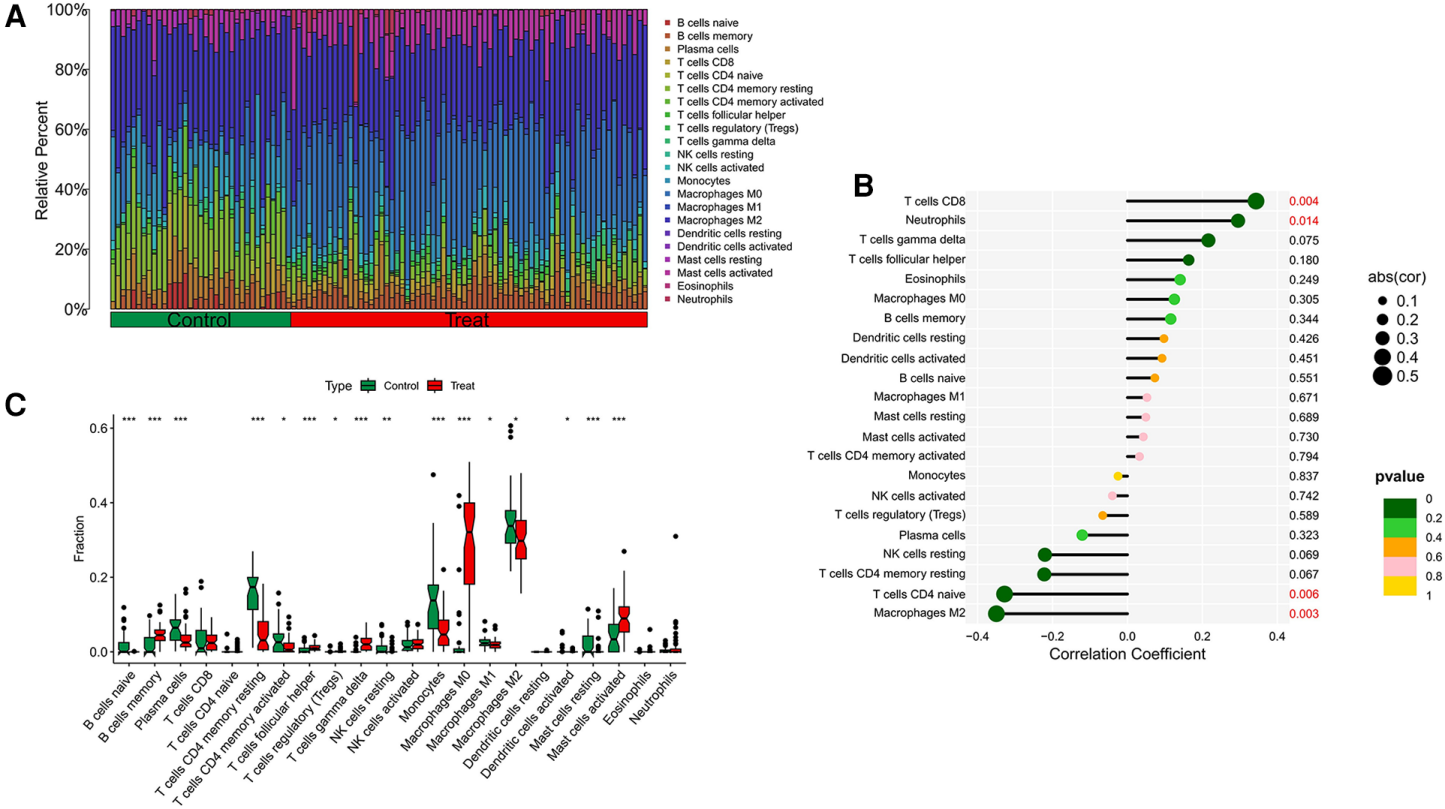
Immune infiltration analysis of ST8SIA4 gene in aS. (**A**) Histogram of proportion of immune cells. (**B**) Correlation between ST8SIA4 and immune cells content. (**C**) Proportion of 22 kinds of immune cells in a boxplot diagram. *p < 0.05; **p < 0.01;***p < 0.001.

**Figure 9:**
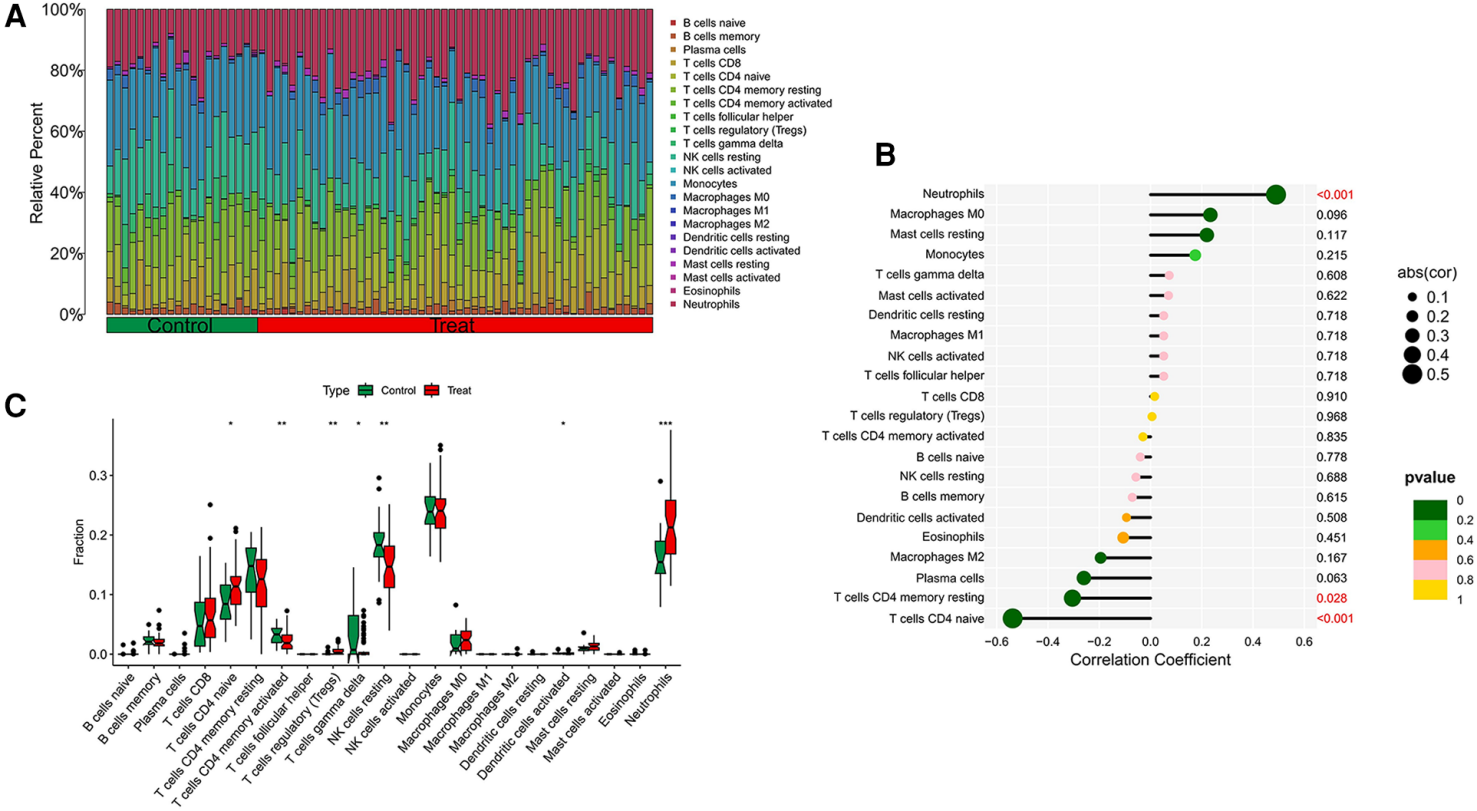
Immune infiltration analysis of ST8SIA4 gene in ankylosing spondylitis. (**A**) Histogram of proportion of immune cells. (**B**) Correlation between ST8SIA4 and immune cells content. (**C**) Proportion of 22 kinds of immune cells in a boxplot diagram. *p < 0.05; **p < 0.01;***p < 0.001.

Our research further delved into the association between the ST8SIA4 gene and the immune system's cellular composition by evaluating the prevalence of 22 distinct immune cell types within samples from AS and ankylosing spondylitis (Figures 8A, 9A). The AS samples displayed increased levels of several immune cells, including B cells memory, T cells follicular helper, T cells regulatory, T cells gamma delta, macrophages M0, and mast cells activated. Conversely, the same samples showed decreased quantities of B cells naive, plasma cells, T cells CD4 memory resting and activated, NK cells resting, monocytes, macrophages M1 and M2, and mast cells resting (Figure 8C). In comparison to healthy controls, samples from ankylosing spondylitis cases notably had higher levels of T cells CD4 naive, T cells regulatory, and neutrophils, but lower levels of T cells CD4 memory activated, T cells gamma delta, and NK cells resting (Figure 9C). Furthermore, we observed a positive correlation between the ST8SIA4 gene expression and the presence of T cells CD8 and neutrophils, and a negative correlation with macrophages M2 and T cells CD4 naive in AS samples (Figure 8B). Similarly, in ankylosing spondylitis, there was a positive correlation with neutrophil levels and a negative correlation with T cells CD4 naive and T cells CD4 memory resting (Figure 9B). These findings, which are statistically significant with a *P*-value <0.05, clarify the influence of ST8SIA4 on immune cell dynamics.

### 3.7 Expression of ST8SIA4 in single cells

**Figure 10:**
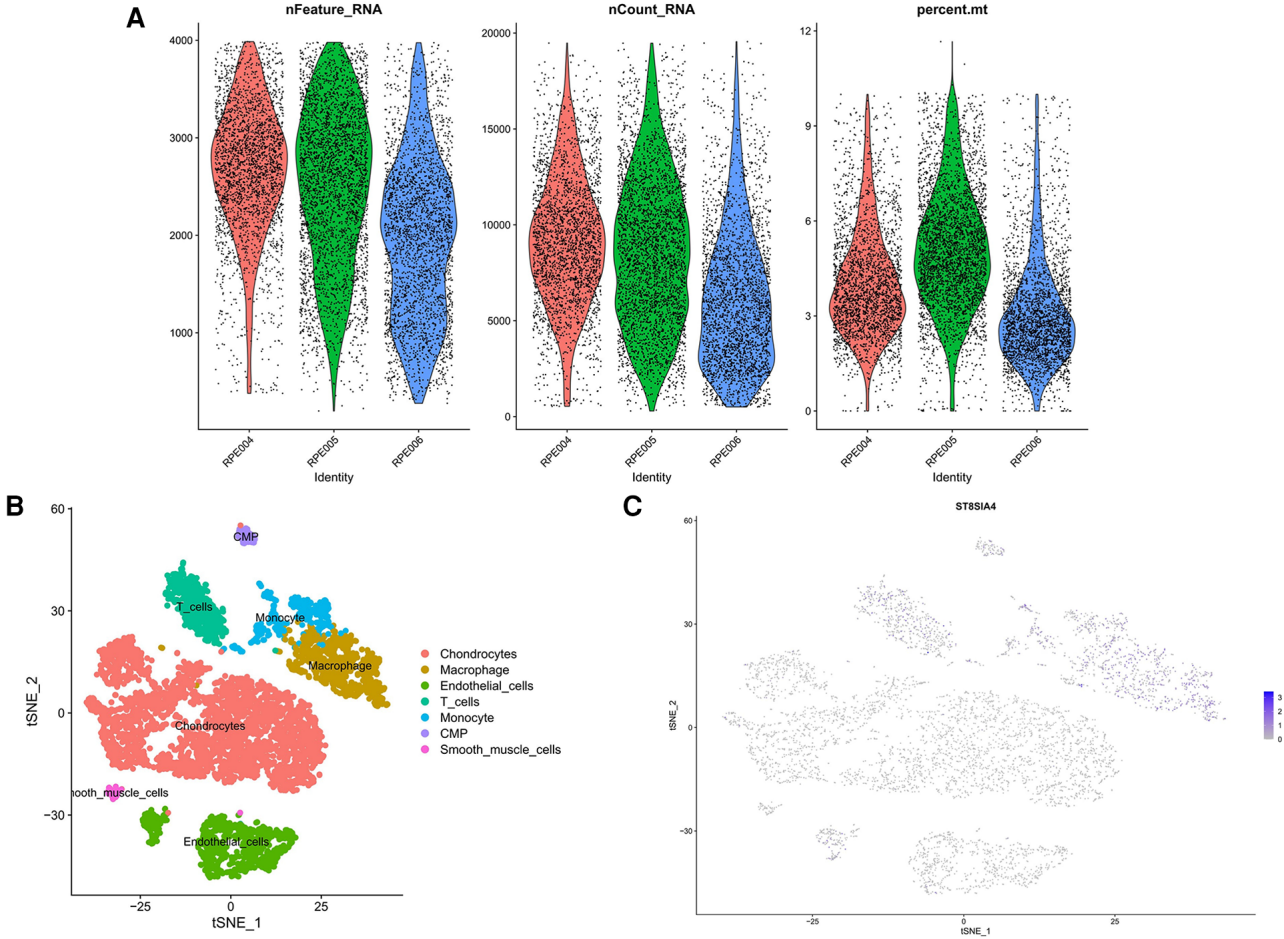
Quality control results for single-cell data. The number of genes, the number of gene reads and the proportion of mitochondrial genes were represented in order from top to bottom. (**A**) The panel was post-processing. (**B**) Cellular subtypes of AS. (**C**) Scatter plot of the expression of ST8SIA4.

We retrieved single-cell data from the GSE155512 dataset and performed analysis using the Seurat toolkit, applying the t-SNE algorithm for clustering cells. After quality control measures, cells that did not meet quality standards were excluded (Figures 10A). Cells from three distinct samples were categorized into seven subgroups: Chondrocytes, Macrophages, Endothelial Cells, T Cells, Monocytes, CMP, and Smooth Muscle Cells (Figure 10B). Further analysis indicated that the genes ST8SIA4 was predominantly expressed in Macrophages, Monocytes, T Cells, and CMP (Figure 10C).

## 4 Discussion

Both AS and ankylosing spondylitis involve inflammatory responses and abnormal immune system activation. Studies suggest that AS development commences with endothelial cell damage and dysfunction. This damage facilitates the recruitment of inflammatory cells, especially monocytes and T cells, into the subendothelial space. The activated cells contribute to the accumulation of oxidized low-density lipoprotein (LDL) and foam cell formation, which are pivotal in plaque development within vascular walls. Released cytokines and enzymes from these cells during plaque formation accelerate its growth and complexity, eventually leading to vascular narrowing or thrombosis (15, 27). In ankylosing spondylitis, inflammation primarily affects the spine and sacroiliac joints, involving chronic immune cell activation and continuous inflammatory mediator release. A critical pathway in this condition is the activation of the IL-23/IL-17 axis, which promotes the proliferation of Th17 cells and increased expression of IL-17. This cytokine not only stimulates inflammatory cell recruitment and activation but also influences bone metabolism, contributing to pathological bone formation (28–30). Additionally, the IL-23/IL-17 axis in AS is linked to vascular inflammation and diminished plaque stability (31). Moreover, both conditions share key aspects of immune regulation, notably elevated T-cell-mediated responses and inflammatory cytokines like TNF*α*, IL-6, and IL-1β, reflecting an increased cardiovascular disease risk in ankylosing spondylitis patients (4, 18, 32). The extensive inflammatory state may predispose these individuals to cardiovascular conditions, akin to the chronic inflammation observed in AS. Despite affecting different physiological systems, the similarities in their inflammatory pathways and immune dysregulation underscore potential links between these conditions, providing valuable insights for novel therapeutic approaches focusing on inflammation and immune regulation. Further research is imperative to explore these pathways more comprehensively, aiming to devise new treatment strategies and enhance patient outcomes.

The exact mechanisms underlying both AS and ankylosing spondylitis remain elusive. This study investigates their common pathways and biomarkers through bioinformatics analysis. RNA-seq profiles from the GEO database were analyzed to compare AS and ankylosing spondylitis. Employing the SVM-RFE algorithm, we identified key core genes, notably ST8SIA4, and elucidated a shared lysosomal pathway in both diseases. The potential of ST8SIA4 as a diagnostic biomarker for AS and ankylosing spondylitis was further validated. Analysis of immune infiltration profiles revealed elevated levels of regulatory T cells in the disease cohorts, suggesting a shared immunological basis in the pathogenesis of AS and ankylosing spondylitis.

T cells regulatory (Tregs) comprise a specific group of cells recognized for their ability to modulate immune responses. They achieve this by restraining the proliferation, activation, and secretion of inflammatory cytokines from effector T cells (Th) through mechanisms such as the release of suppressive cytokines (IL-10, TGF-β) and through direct cell-to-cell contact inhibition. This action maintains self-tolerance and mediates anti-inflammatory effects (33). In AS, arterial wall inflammation is driven by adaptive and innate immune responses (34–36). Tregs mitigate this inflammation by curbing pro-inflammatory responses from Th1/Th17 cells and reducing antigen presentation by dendritic cells (37–39). Furthermore, Tregs suppress cytokine secretion, inhibit macrophage-driven inflammation, and regulate cholesterol metabolism and foam cell formation (40). Studies have shown that reduced Treg numbers within atherosclerotic plaques correlate with increased plaque vulnerability and rupture, whereas increased Treg levels can slow AS progression (41–43). Research by Nilsson J et al. suggests that Tregs control autoreactive T cell activity to prevent AS development (44). Similarly, animal models have demonstrated Treg responsiveness to apolipoprotein B100-derived peptides, reducing AS incidence and progression (45, 46). Conversely, Treg depletion exacerbates AS in hypercholesterolemic mice (38, 39). These findings underscore Tregs' crucial role in managing AS occurrence and progression. Moreover, studies indicate Treg functional impairments in ankylosing spondylitis, potentially due to IL-2 deficiencies, reduced STAT5 phosphorylation, decreased FOXP3 expression, and increased CpG methylation in the CNS2 region of the FOXP3 gene. These impairments lead to uncontrolled effector CD4+ T cell proliferation and immune imbalance (47, 48). Liao et al. found that increased Treg numbers in ankylosing spondylitis patients correlate positively with disease activity, supporting a feedback mechanism where inducible Tregs arise from persistent inflammation (49–51). Continued research is essential to elucidate the mechanisms by which Tregs influence AS and ankylosing spondylitis development.

This study identifies ST8SIA4 as a key diagnostic marker in the progression of AS and ankylosing spondylitis, noting a significant increase in its expression in affected patients. ST8SIA2 and ST8SIA4, two polysialyltransferases located in the Golgi apparatus, facilitate the addition of polysialic acid chains to proteins, including themselves via autopolysialylation (52). Notably, these enzymes are significant for polysialylating the Neural Cell Adhesion Molecule (NCAM) specifically at its fifth Ig domain, which leads to the formation of polymers that include 60–90 sialic acid residues (53, 54). The introduction of this extensive, negatively charged side chain is crucial as it interferes with PSA-NCAM binding interactions, subsequently promoting enhanced cell motility (55). Despite these insights, the functional mechanisms of ST8SIA4 remain unclear. Shu et al. have established that increased St8sia4 expression correlates positively with the presence of MDSCs, macrophages, and Treg cells. This association suggests that St8sia4 plays a pivotal role in altering the tumor microenvironment, thereby facilitating tumor growth and metastasis (56). In this research, we observed that in AS patients, ST8SIA4 correlates positively with neutrophil and CD8+ T-cell levels, but negatively with naïve CD4T cells and M2 macrophages. In cases of ankylosing spondylitis, similar positive correlations with neutrophils and negative correlations with both naïve and resting memory CD4T cells were noted. Although direct evidence linking ST8SIA4 to these diseases is lacking, the data suggest its involvement in regulating cell adhesion, immune responses, and inflammation in vascular tissues, thereby potentially influencing the pathogenesis of AS and ankylosing spondylitis through complex immune and inflammatory mechanisms, warranting further investigation (56, 57).

Lysosomes serve as crucial regulators of cellular and organismal homeostasis, mediating essential processes such as signal transduction, cellular metabolism, proliferation, differentiation, secretion, and the quality control of proteins and organelles (58, 59). Studies have identified a significant role for lysosomes in cholesterol metabolism; dysfunction in these organelles can result in cholesterol accumulation within cells (60, 61), contributing to foam cell formation and atherosclerotic plaque development. Additionally, lysosomes facilitate the removal of cellular debris, including damaged proteins and organelles, via the autophagy pathway (62, 63). Autophagy aids in the hydrolysis of lipid droplets and the efflux of free cholesterol from foam cells, thereby playing a protective role against AS (64). Conversely, lysosomal dysfunction can impair autophagic processes, leading to the buildup of cellular waste, which in turn promotes inflammatory responses and AS progression (60). Furthermore, abnormalities in lysosomes can lead to the activation of the NLRP3 inflammasome, which initiates the secretion of pro-inflammatory cytokines like IL-1β and IL-18 (65–67). These cytokines are instrumental in enhancing the recruitment and activation of inflammatory cells, including macrophages and T cells (58, 68), and in increasing the expression of cytokines, chemokines, and adhesion molecules (69, 70). This cascade amplifies inflammatory responses and exacerbates the progression of AS.

The lysosomal pathway is intricately linked to the pathogenesis of ankylosing spondylitis. Research has demonstrated that the disease-associated HLA-B27 subtype exhibits increased resistance to lysosomal degradation, potentially leading to a prolonged immune response and contributing to AS pathogenesis (71). Additionally, lysosomes play a pivotal role in autoimmune diseases like AS by modulating cell death, autophagy, inflammasome-related cytokines, and various metabolic pathways, including sphingolipid metabolism (58). This investigation additionally recognizes the lysosomal pathway as a plausible shared mechanism contributing to the development of AS, highlighting the necessity for ongoing investigation into these interconnected pathomechanisms.

The focus of this paper is the investigation of shared immune pathways, pivotal genes, and immune infiltration characteristics of AS. This retrospective study recognizes the need for external validation and has validated key genes across multiple datasets. Future research will aim to verify the function of these genes both *in vitro* and *in vivo*. The consistency with previous studies enhances this research's reliability. Subsequent efforts will focus on the collection of peripheral blood specimens, performing RT-qPCR analysis on essential genes, and executing cohort studies to assess the gene expression in patients with combined AS and ankylosing spondylitis, their linkage to cardiovascular incidents, and their prognostic value.

## 5 Conclusion

In this study, we employed bioinformatics techniques and machine learning algorithms to identify ST8SIA4 as a shared diagnostic gene for AS and ankylosing spondylitis. We additionally confirmed the diagnostic utility of ST8SIA4 through an independent dataset. Furthermore, CIBERSORT analysis showed a correlation between the expression of ST8SIA4 and the infiltration of immune cells. Single-cell sequencing analysis reveals that ST8SIA4 is primarily expressed in macrophages, monocytes, T cells, and CMP. GSEA analysis indicated that the lysosomal pathway is enriched across both examined conditions. This investigation supports the potential of ST8SIA4 as a diagnostic biomarker and aids in exploring the common mechanisms associated with AS and ankylosing spondylitis. Future research is directed towards understanding the function of the lysosomal pathway and the effects of ST8SIA4 via both *in vitro* and *in vivo* studies.

## Data Availability

The original contributions presented in the study are included in the article/Supplementary Material, further inquiries can be directed to the corresponding author.
