# Supplementary material for: Exploring the common mechanisms and biomarker ST8SIA4 of atherosclerosis and ankylosing spondylitis through bioinformatics analysis and machine learning
Source: Front Cardiovasc Med. 2024 Jul 18;11:1421071. doi: 10.3389/fcvm.2024.1421071 (PMC11310936; doi:10.3389/fcvm.2024.1421071)
Supplement: Supplementary file 2 [file Datasheet1.pdf]

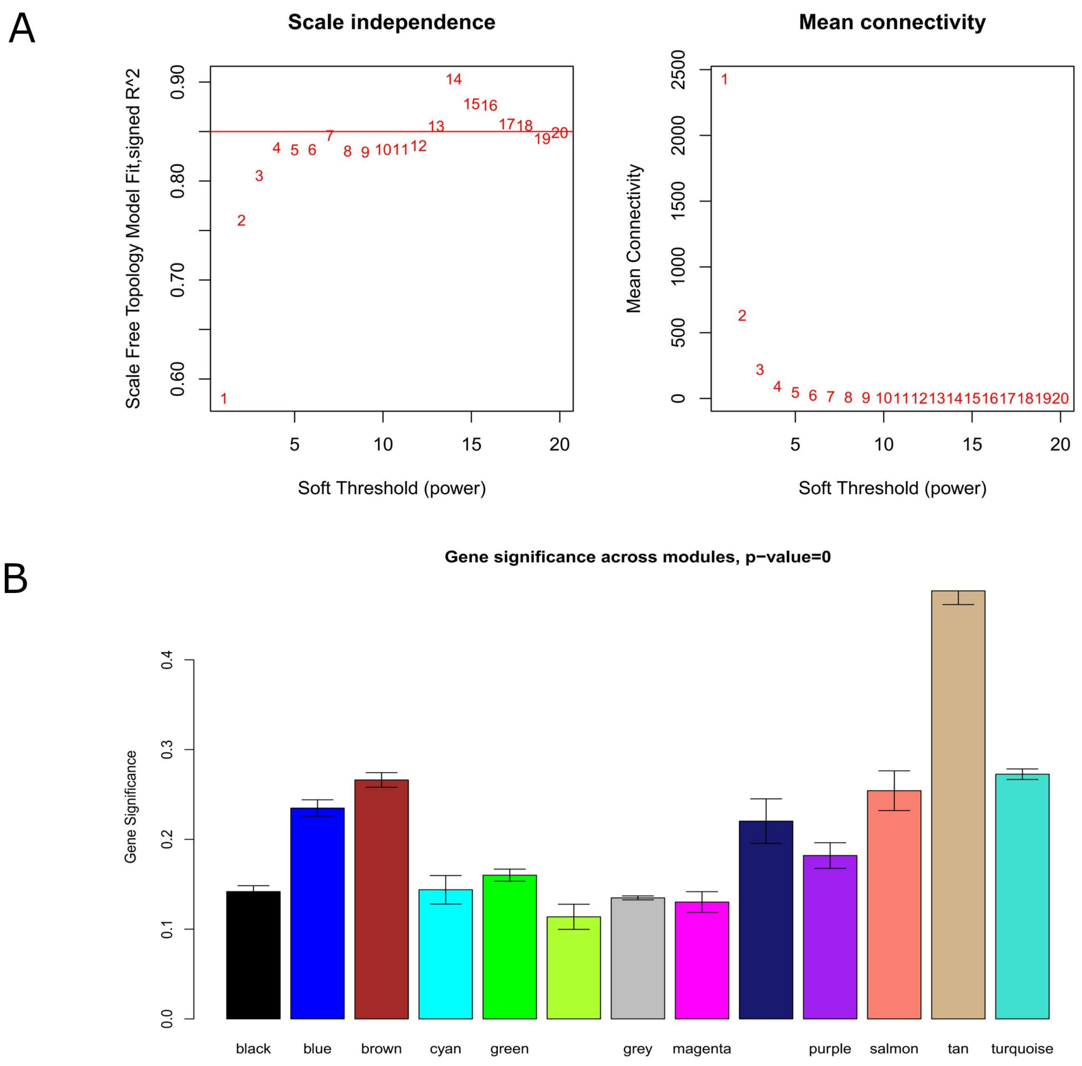

Supplementary File 1: **(A)** The determination of soft thresholding power in GSE73754. **(B)** The gene significance across modules in GSE73754.
