# Supplementary material for: Exploring the common mechanisms and biomarker ST8SIA4 of atherosclerosis and ankylosing spondylitis through bioinformatics analysis and machine learning
Source: Front Cardiovasc Med. 2024 Jul 18;11:1421071. doi: 10.3389/fcvm.2024.1421071 (PMC11310936; doi:10.3389/fcvm.2024.1421071)
Supplement: Supplementary file 3 [file Datasheet2.docx]

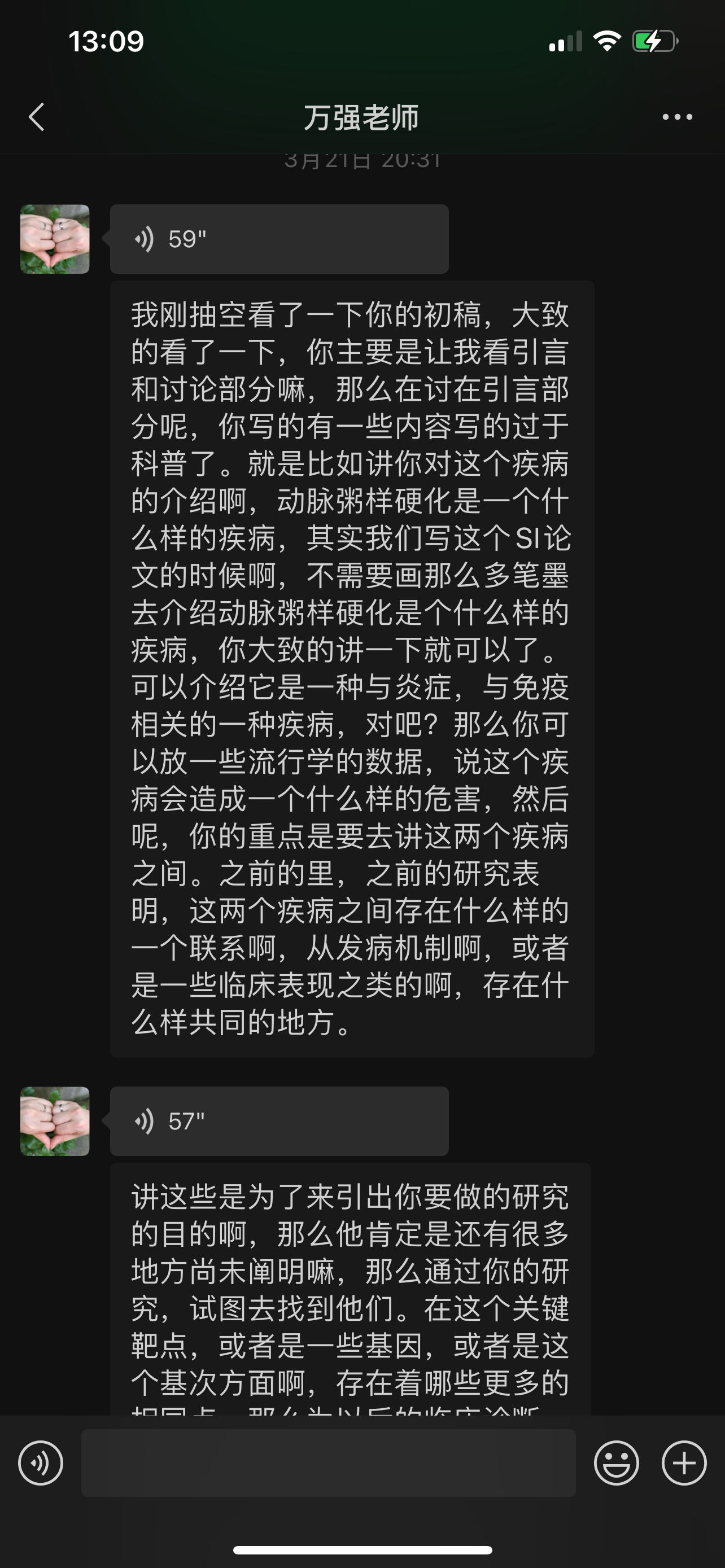


I just had a moment to look over your draft briefly. You wanted me to focus on the introduction and the discussion sections, right? In the introduction part, some of the content is too much like popular science. For instance, you talk about the disease introduction, what atherosclerosis is. Actually, when writing this paper, we don’t need to spend so much ink explaining what atherosclerosis is. A brief overview would suffice. You could mention that it is a disease related to inflammation and immunity, right? Then, you can include some epidemiological data, discussing the kind of harm this disease causes. After that, the focus should be on the link between these two diseases. Previous research has shown what kind of connections exist between them, whether in terms of pathogenesis or clinical manifestations, highlighting what they have in common.

Discussing these aspects is intended to lead into your research aims. Obviously, there are still many areas that remain unclear, so your study aims to explore them.


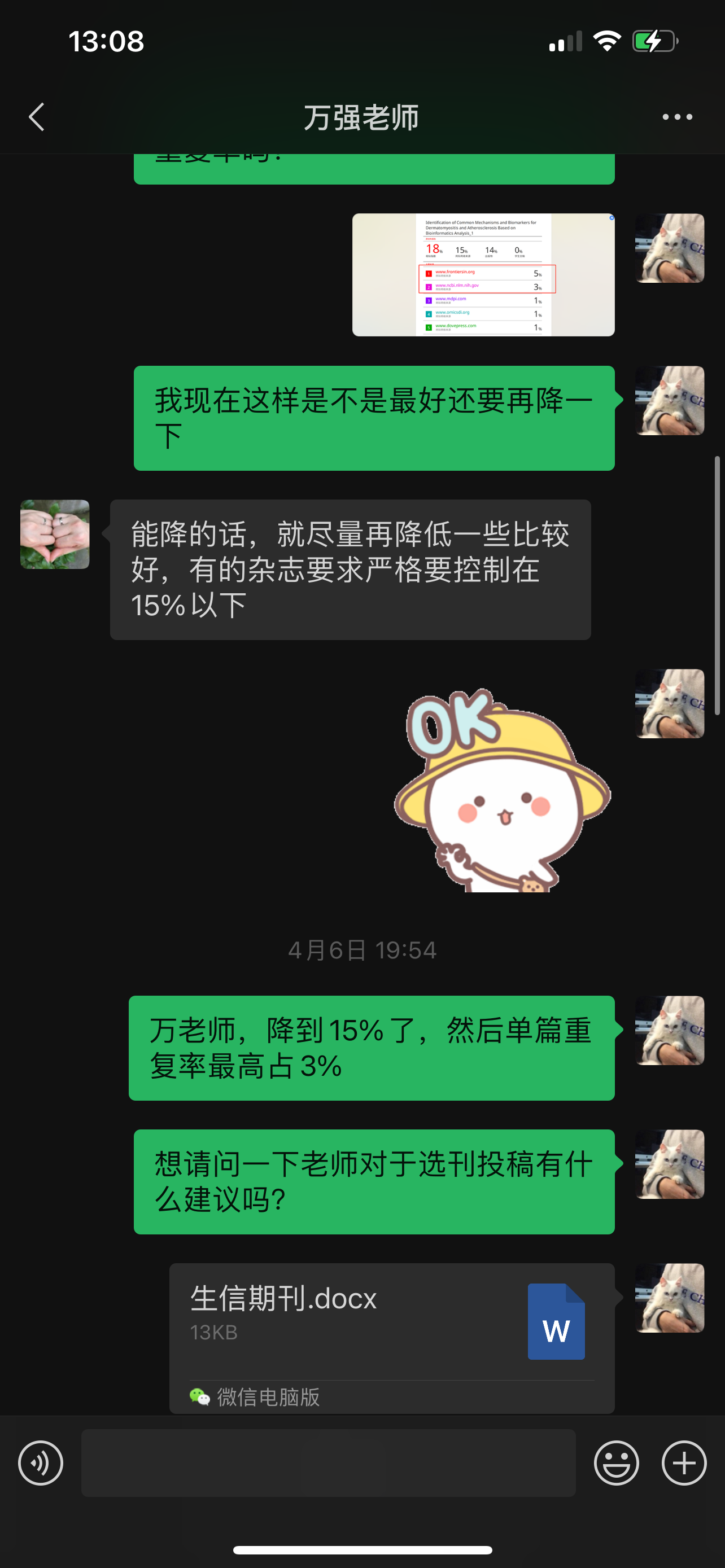


Mr. Wan, it's down to 15%.

The maximum review rate of a single article is 3%.

I'd like to ask you if you have any suggestions on how to choose a journal for submission?

Would it be better if I dropped a little more?

If you can, try to lower it a bit.Some magazines require a strict limit of 15% or less.


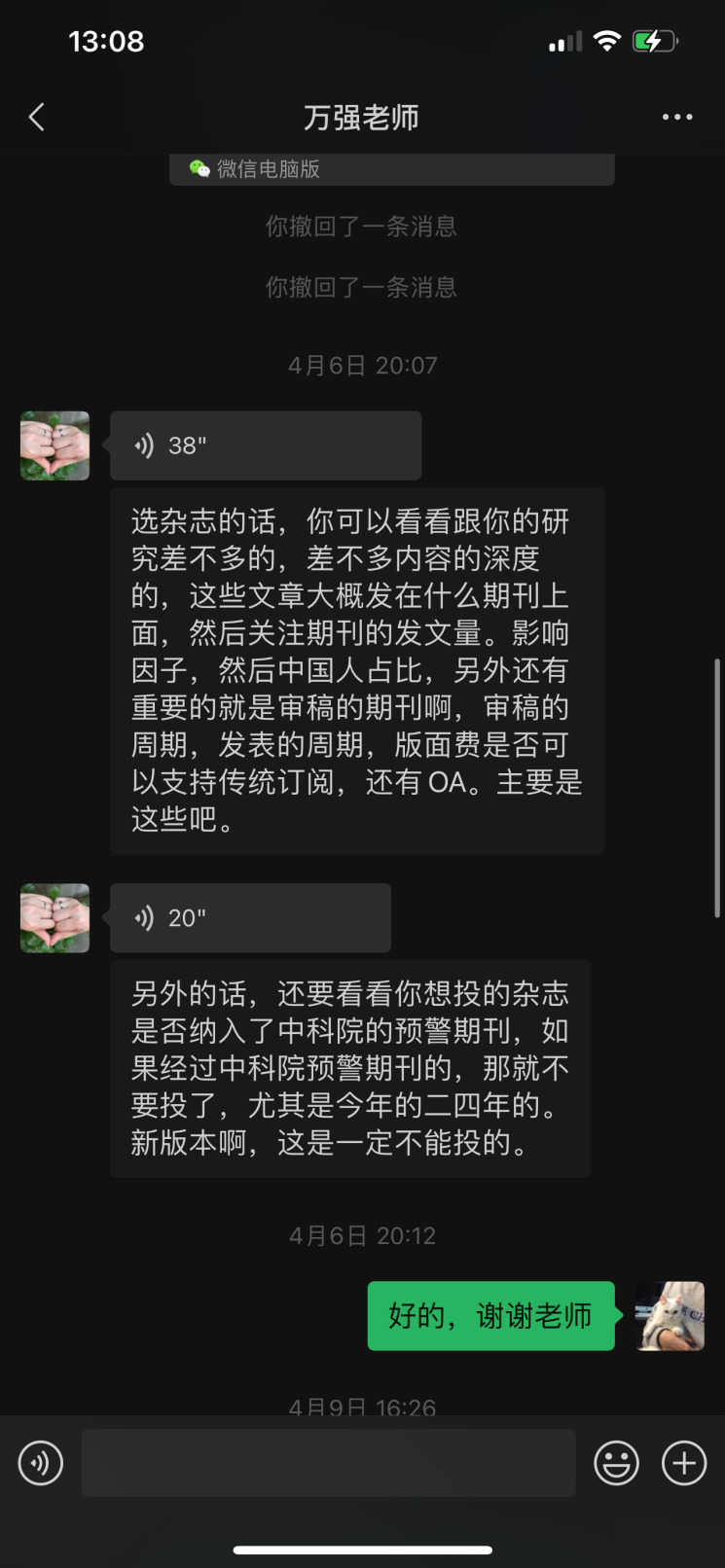


Additionally, you should check if the journal you are considering is listed in the Chinese Academy of Sciences’warning journals. If it has been flagged by them, particularly in this year's 2024 update, you definitely should not submit your work there.

When choosing a journal, you should look at those that publish research similar to yours, with similar content depth. See where such articles are typically published, then consider the journal's publication volume, impact factor, and the proportion of contributions from Chinese researchers. Additionally, pay attention to important aspects like the journal's review period, publication timeline, whether page charges are covered under traditional subscriptions, and the availability of Open Access (OA) options. These are the main factors to consider.


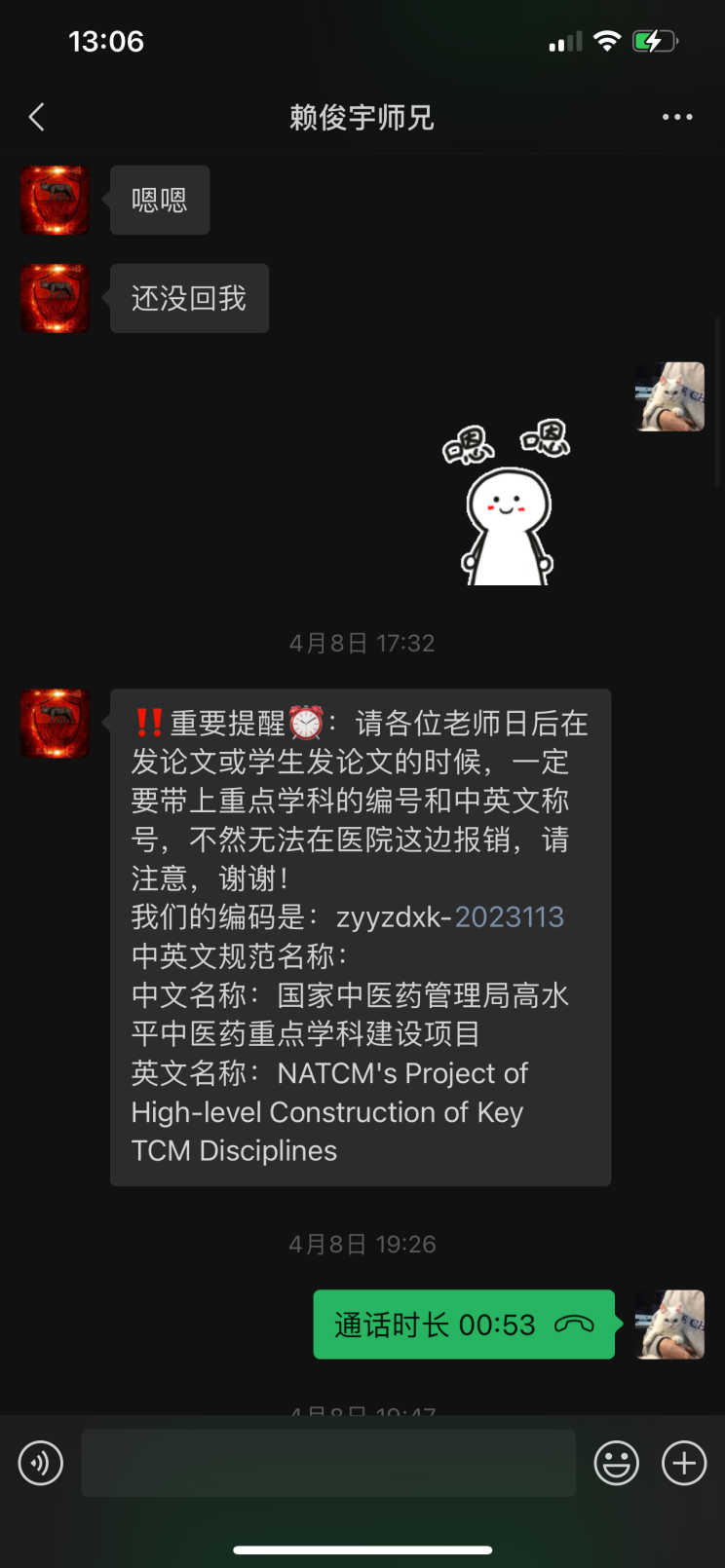


Important Reminder: In the future, when faculty members or students submit papers, please make sure to include the key discipline code along with its name in both Chinese and English. Otherwise, you will not be able to get reimbursed by the hospital. Thank you!

Our code is: zyyzdxk-2023113

Standard names are:

Chinese Name: 国家中医药管理局高水平中医药重点学科建设项目

English Name: NATCM's Project of High-level Construction of Key TCM Disciplines


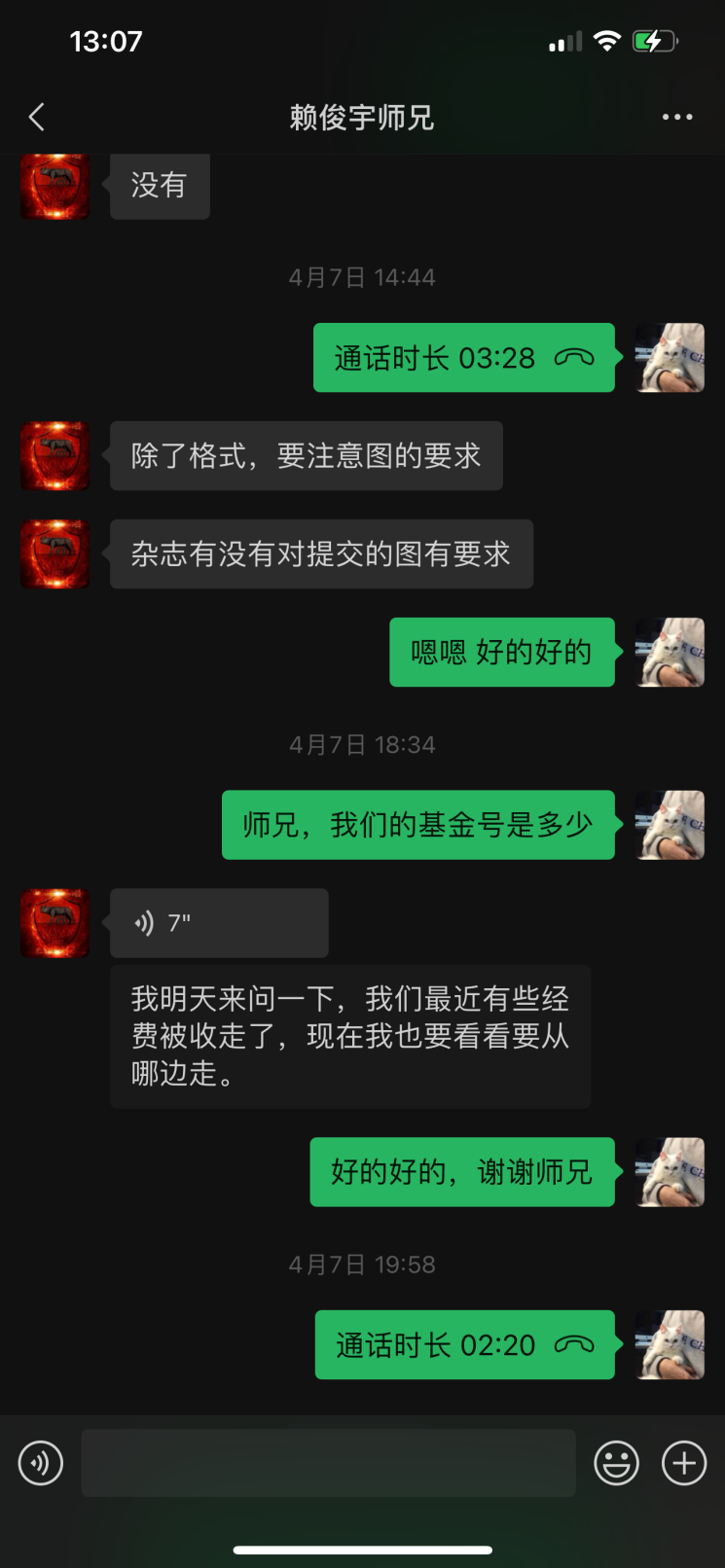


In addition to the format, be mindful of the requirements for figures. Does the journal have specific requirements for the images you submit?

Alright, got it.


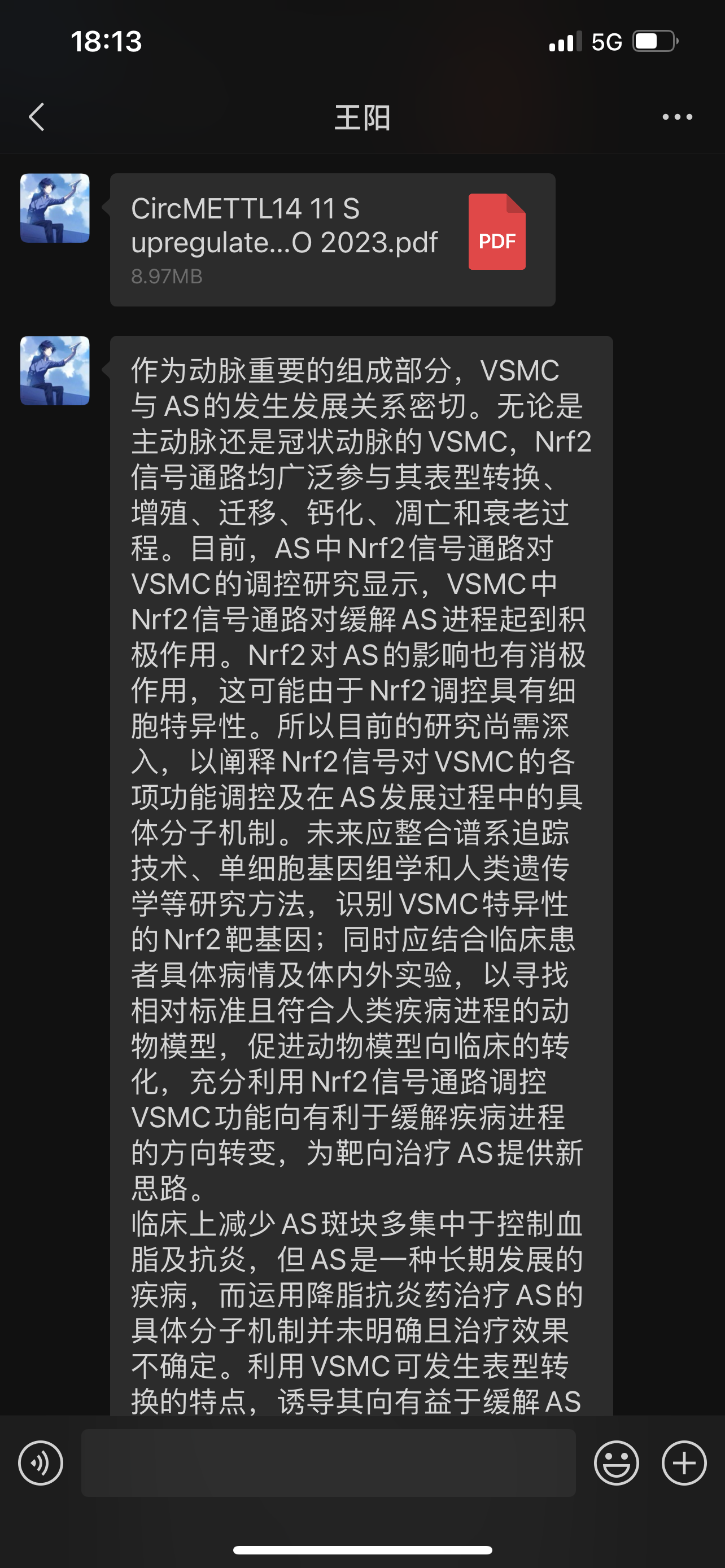


As an important component of arteries, Vascular Smooth Muscle Cells (VSMCs) are closely related to the development of Atherosclerosis (AS). Whether in the aorta or coronary arteries, the Nrf2 signaling pathway extensively participates in their phenotypic transformation, proliferation, migration, calcification, apoptosis, and aging processes. Current research on the regulation of VSMCs by the Nrf2 signaling pathway in AS shows that the Nrf2 pathway plays a positive role in alleviating the progression of AS. However, Nrf2 also has negative effects on AS, which may be due to the cell-specific regulation by Nrf2. Therefore, current studies need to delve deeper to elucidate the regulatory functions of Nrf2 signaling on VSMCs and the specific molecular mechanisms in the development of AS. Future research should integrate lineage tracing, single-cell genomics, and human genetics to identify VSMC-specific Nrf2 target genes; it should also combine specific clinical conditions and in vivo and in vitro experiments to find relatively standard and human disease-relevant animal models. This will promote the translation of animal models to clinical settings and maximize the utilization of the Nrf2 signaling pathway in controlling disease progression.


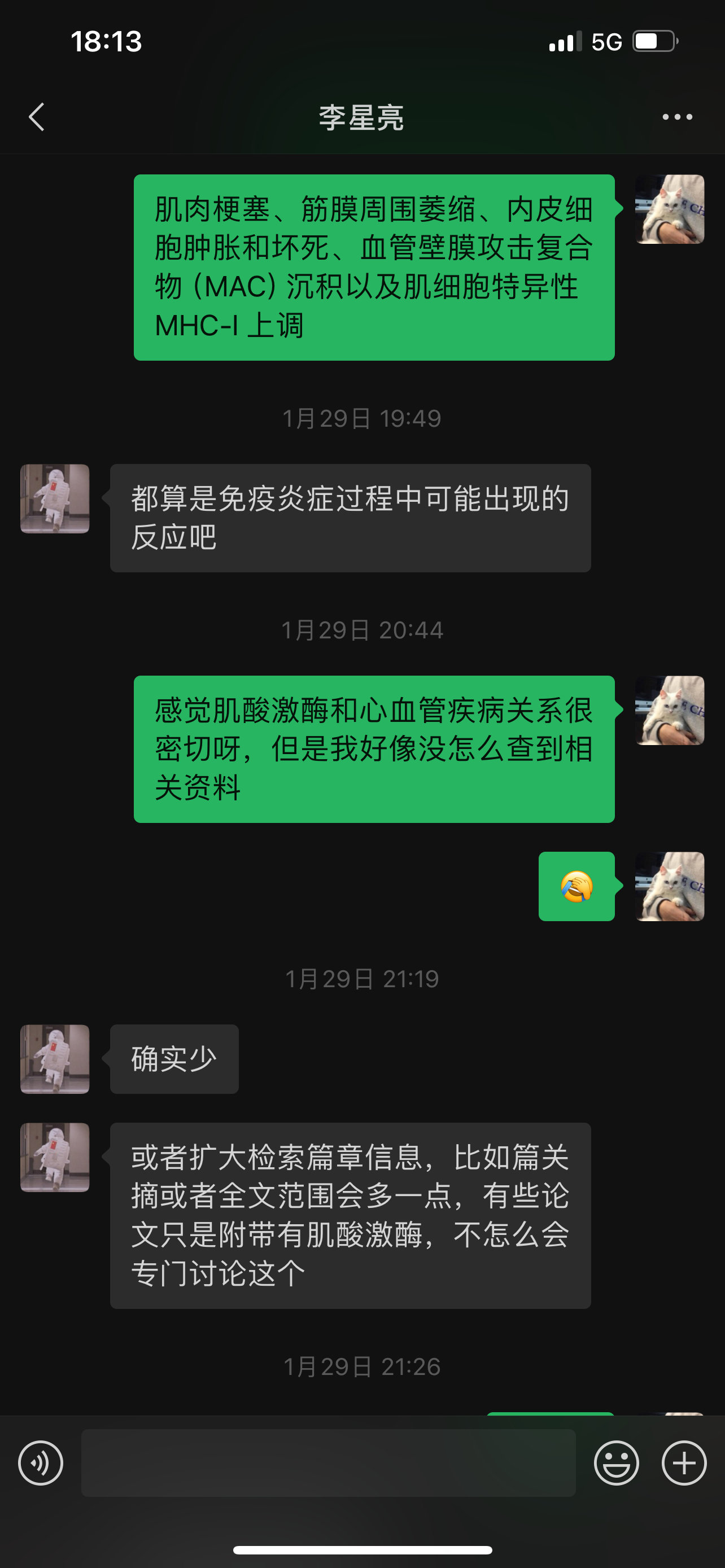


Or perhaps expand the search scope to include abstracts or full texts, which might yield more results. Some papers only mention creatine kinase incidentally and do not focus on discussing it specifically.

It seems that there is a close relationship between creatine kinase and cardiovascular diseases, but I haven't found much related information.

These are all reactions that may occur during the immune-inflammatory process.

Muscle infarction, perifascial atrophy, endothelial cell swelling and necrosis, deposition of the membrane attack complex (MAC) in the vascular wall, and upregulation of muscle cell-specific MHC-1.


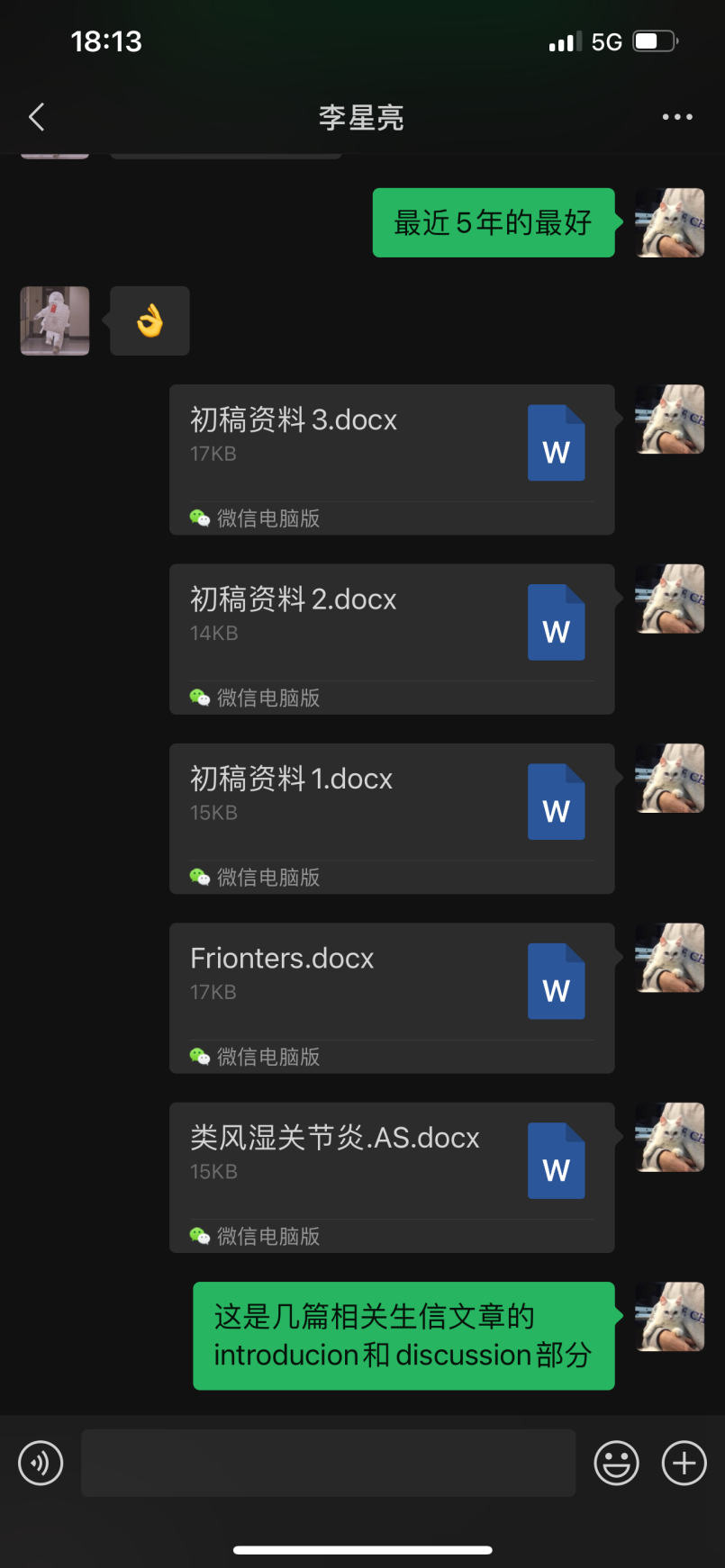


These are the introduction and discussion sections of several related bioinformatics articles.


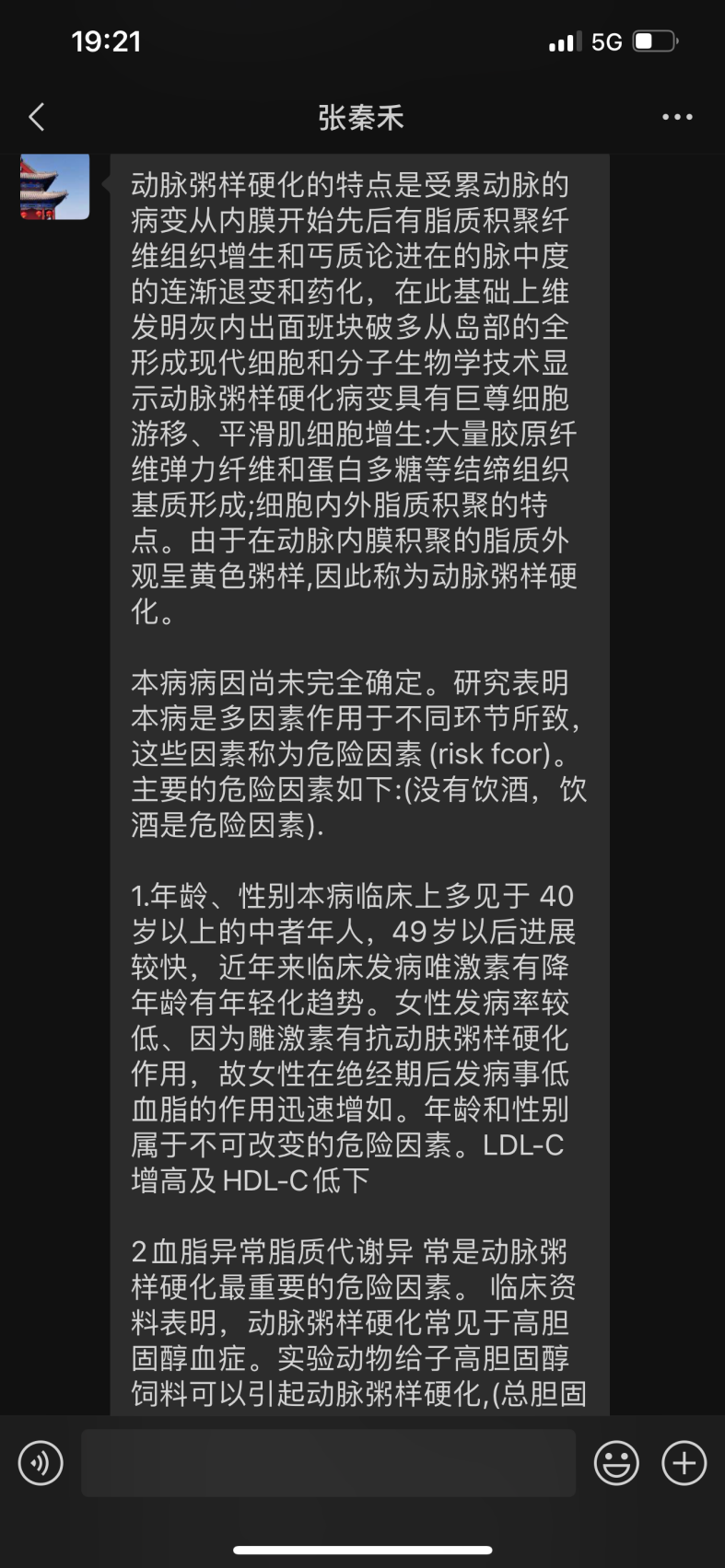


The characteristics of atherosclerosis involve the affected arteries undergoing pathological changes starting from the intima, with successive stages of lipid accumulation, fibrous tissue proliferation, and calcification, leading to moderate degeneration and calcification within the arteries. On this basis, plaques often rupture, leading to thrombosis originating from the site of the plaque. Modern cellular and molecular biology techniques have shown that atherosclerotic lesions are characterized by the migration of macrophages, proliferation of smooth muscle cells, and the formation of a connective tissue matrix composed of abundant collagen fibers, elastic fibers, and proteoglycans; there is also characteristic intra- and extracellular lipid accumulation. The lipid accumulation within the arterial intima appears yellow and mushy, hence the term "atherosclerosis."

The exact causes of this disease have not yet been fully determined. Research indicates that the disease is caused by multiple factors acting at different stages, known as risk factors. The main risk factors include the following: (non-drinking, as drinking is a risk factor).


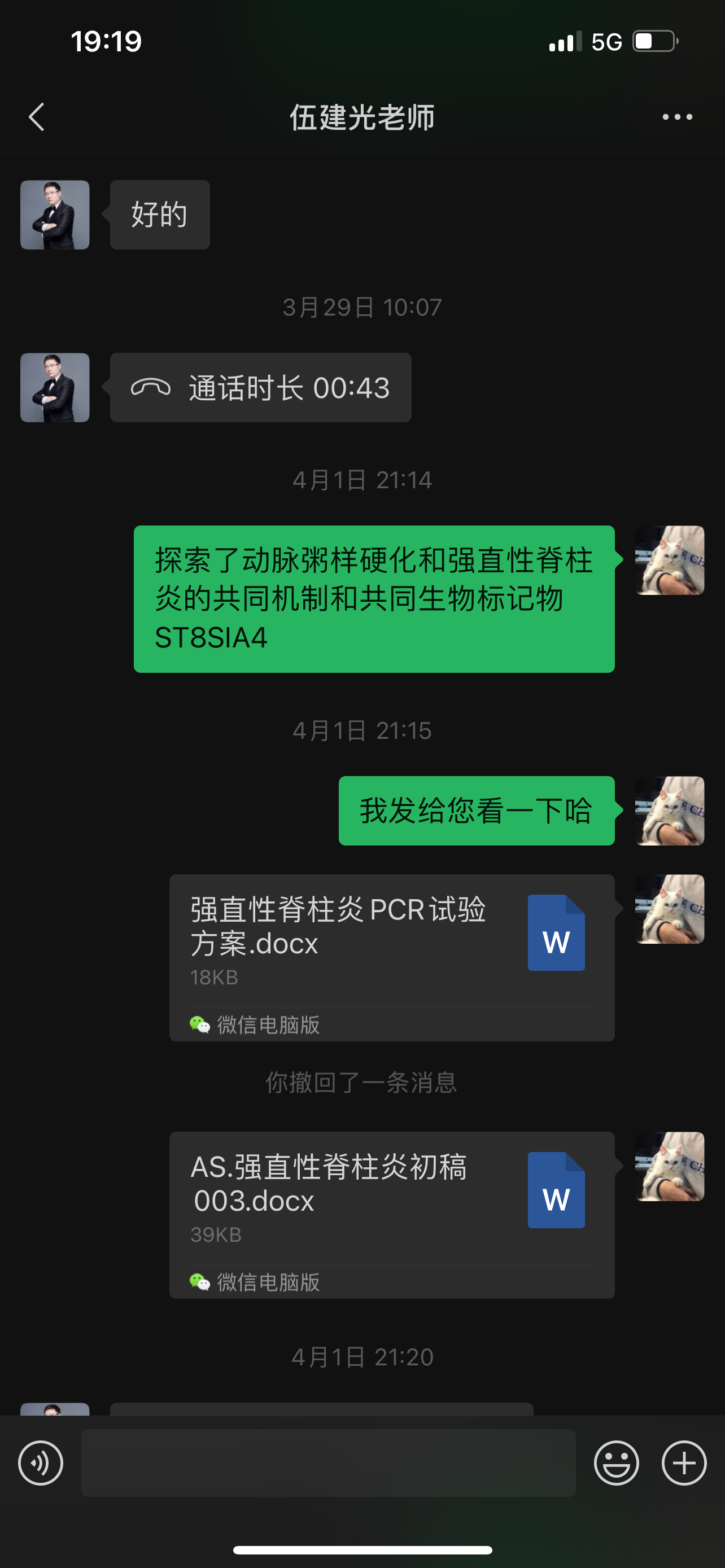


Exploring the common mechanisms and biomarker ST8SIA4 of atherosclerosis and ankylosing spondylitis through bioinformatics analysis and machine learning

I'll send it to you.
