# Supplementary material for: Exploring the common mechanisms and biomarker ST8SIA4 of atherosclerosis and ankylosing spondylitis through bioinformatics analysis and machine learning
Source: Front Cardiovasc Med. 2024 Jul 18;11:1421071. doi: 10.3389/fcvm.2024.1421071 (PMC11310936; doi:10.3389/fcvm.2024.1421071)
Supplement: Supplementary file 4 [file Datasheet3.pdf]

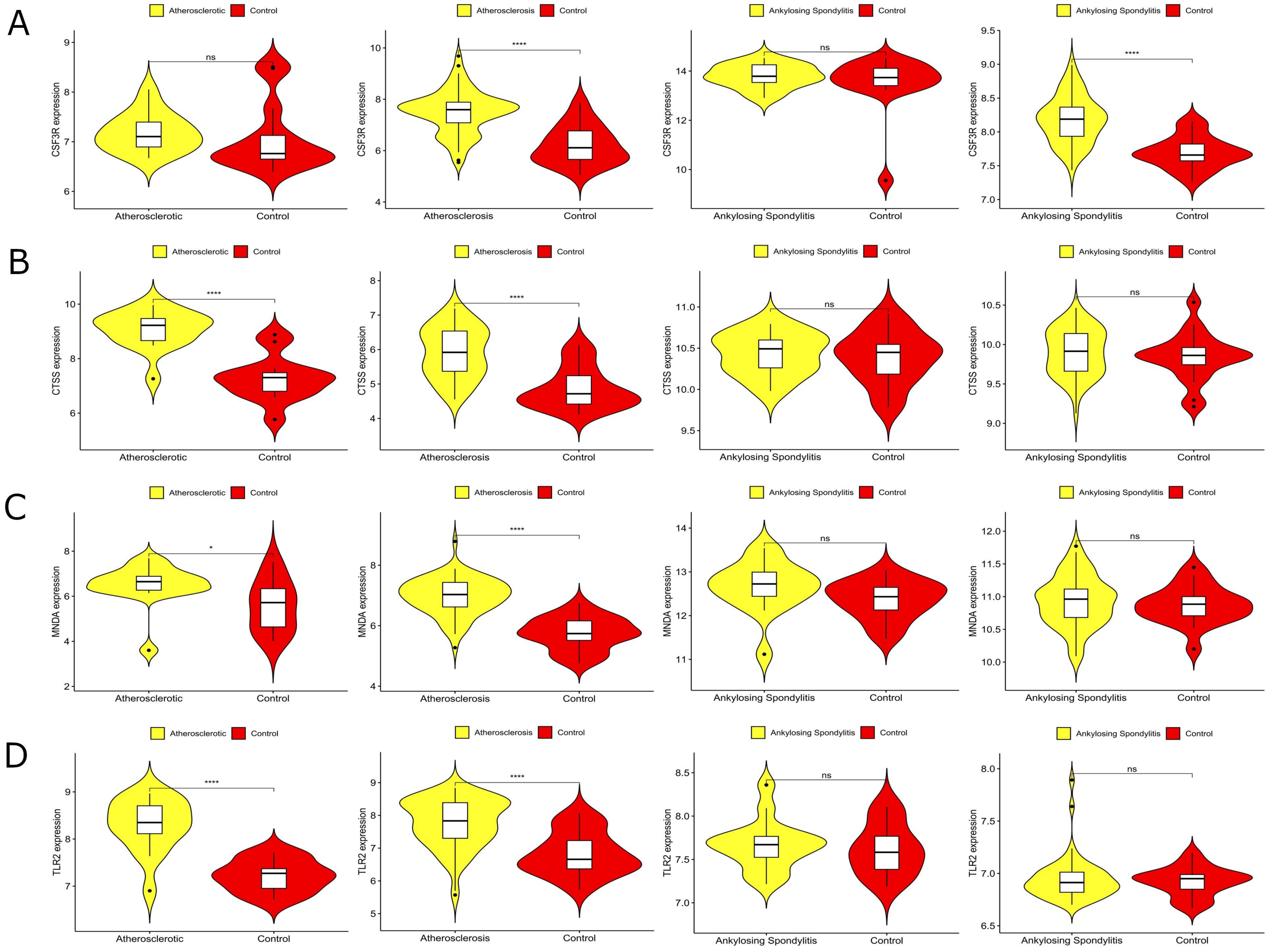

Supplementary File 3: Validation of the expression level of CSF3R, CTSS, MNDA, TLR2. **(A)** The violin plots of CSF3R in GSE28829, GSE100927, GSE25101, GSE73754. **(B)** The violin plots of CTSS in GSE28829, GSE100927, GSE25101, GSE73754. **(C)** The violin plots of MNDA in GSE28829, GSE100927, GSE25101, GSE73754. **(D)** The violin plots of TLR2 in GSE28829, GSE100927, GSE25101, GSE73754. *p <0.05; **p <0.01;***p <0.001; ****p <0.0001.
